# The Changing Landscape of Doctoral Education in Science, Technology, Engineering, and Mathematics: PhD Students, Faculty Advisors, and Preferences for Varied Career Options

**DOI:** 10.3389/fpsyg.2021.711615

**Published:** 2021-12-17

**Authors:** David K. Sherman, Lauren Ortosky, Suyi Leong, Christopher Kello, Mary Hegarty

**Affiliations:** ^1^Department of Psychological & Brain Sciences, University of California, Santa Barbara, CA, United States; ^2^Merced Psychological Sciences, University of California, Merced, CA, United States

**Keywords:** graduate education, professoriate, diversity, norms, STEM education and careers

## Abstract

The landscape of graduate science education is changing as efforts to diversify the professoriate have increased because academic faculty jobs at universities have grown scarce and more competitive. With this context as a backdrop, the present research examines the perceptions and career goals of advisors and advisees through surveys of PhD students (Study 1, *N* = 195) and faculty mentors (Study 2, *N* = 272) in science, technology, engineering, and math disciplines. Study 1 examined actual preferences and career goals of PhD students among three options: research careers, teaching careers, and non-academic careers in industry, and compared the actual preferences of students with what they perceived as being the normative preferences of faculty. Overall, students had mixed preferences but perceived that their advisors had a strong normative preference for research careers for them. Moreover, students who ranked research positions as most desirable felt the most belonging in their academic departments. Further analyses revealed no differences in career preferences as a function of underrepresented minority (URM) student status or first-generation (FG) status, but URM and FG students felt less belonging in their academic departments. Study 2 examined faculty preferences for different careers for their advisees, both in general and for current students in particular. While faculty advisors preferred students to go into research in general, when focusing on specific students, they saw their preferences as being closely aligned with the career preference of each PhD student. Faculty advisors did not perceive any difference in belonging between their students as a function of their URM status. Discrepancies between student and faculty perceptions may occur, in part, because faculty and students do not engage in sufficient discussions about the wider range of career options beyond academic research. Supporting this possibility, PhD students and faculty advisors reported feeling more comfortable discussing research careers with each other than either non-academic industry positions or teaching positions. Discussion centers on the implications of these findings for interpersonal and institutional efforts to foster diversity in the professoriate and to create open communication about career development.

## Introduction

“I would feel relatively uncomfortable, mostly because my advisor pushes all of their graduate students to apply for academic research positions because prestige is a value that is highly important to them. If I were to approach my advisor about this (industry or teaching position), and I have before, the response is not dismissive, but they are not completely supportive. I know that they would be disappointed because I would not carry on their academic lineage after they put so much work into my research program.”

-STEM PhD Student, on why one might not feel comfortable discussing industry or teaching positions with an advisor.

“My job is to help my students get to the right place for them. I like it when they go into research positions because it means I’ll continue to see them regularly at conferences—I like my students and having them move into career paths where I likely will not see them again is a personal loss for me—but that is grounded in the deepest and narrowest of my selfish desire to remain connected. But my job is to try to help them get the skills and tools they need to pursue their directions. So if they will need more than research-related skills, I try to help them get those tools.”

-STEM Faculty Advisor on how one helps students prepare for non-academic positions.

The landscape of graduate science education is changing in multiple ways, raising new challenges for students, faculty, and educational institutions. The professoriate has increased ethnic and racial diversity, although not at the same rate as students (Davis and Fry, 2019; National Center for Education Statistics, 2020). Recognizing the importance of a diverse professoriate for training the next generation of scientists, there are increasing efforts to foster greater diversity. The National Science Foundation has a specific program, the Alliances for Graduate Education and the Professoriate, whose goal is to “to increase the number of historically underrepresented minority faculty in STEM (Science, Technology, Engineering, and Mathematics) fields (National Science Foundation, 2021).”

A second change to the landscape of graduate science education relates to the eventual professional pathways that those who earn their doctoral degrees pursue. The expectation has historically been that after obtaining a PhD degree, a student will pursue a tenure-track research-focused academic position. This is no longer the case. In 2017, there were as many PhD holders working in the private sector (42%) as there were in educational institutions (43%; National Center for Engineering and Statistics, 2017; Langin, 2019). And a 2019 international survey of PhD students by the journal *Nature* found that 56% ranked academic positions as the sector of work they would most like to pursue, whereas 28% ranked industry highest.

Within this changing context, graduate students entering PhD programs must figure out how to succeed and, at some point, what the next step will be in their professional journey. To guide their decisions, students are likely to look to the norms and expectations of powerful people in their new environment (Austin, 2002). In PhD programs, the *perceived descriptive norms*, that is, what people believe others do, are inferred by watching the actions of senior graduate students and faculty. People are motivated to conform to the perceived norms of successful and powerful individuals (Cialdini and Trost, 1998). For entering PhD students, those successful and powerful people are their department’s faculty, with particular importance attached to their own graduate advisor. People’s behavior is also impacted by *injunctive* norms, or what they believe important others approve of or believe should be done (Cialdini et al., 1991). Because these norms are not stated explicitly, PhD students and advisors may misperceive each other’s goals and desires.

What underlies this program of research is the possibility that PhD students and their advisors may not have accurate information about the beliefs and goals of each other – and that directly ascertaining that information could be beneficial for communication between them. In this way, the paper is consistent with the central assumptions of social norms interventions expressed by Miller and Prentice (2016, p. 340): “…accurate information about what peers or relevant others think, feel, or do is not always known or salient to people…providing people with this information has the potential to alter their understanding of group norms, their standing in the group, and the evaluative significance of the behavior in question. This altered understanding may, in turn, lead them to act differently.”

Potentially, inaccuracies in perceptions could influence PhD students and advisors alike, affecting their behavior toward each other. The first goal of this research is to identify the actual preferences STEM PhD students have about pursuing different career outcomes – and what they perceive to be the preferences of STEM faculty advisors (Study 1). We focus on three primary career options that students in PhD programs consider: teaching-oriented academic positions, research-oriented academic positions, and non-academic positions (including industry and government). We examine whether there are discrepancies between PhD students’ preferences and their perceptions of their advisors’ preferences for them. We further investigate whether these preferences are related to important outcomes such as perceived support and belonging and how this may be moderated by factors such as underrepresented minority (URM) status or being the first in the family to attend college.

The second goal is to examine the actual normative beliefs that STEM professors have about their students’ career paths. In Study 2, faculty advisors indicate their career preferences for specific students that they are currently advising and their preferences for students they train in general. We also examine the comfort of faculty advisors in discussing different career options.

Together these studies seek to elucidate the dynamic between PhD students and their advisors by examining the perceptions that each has about career development and mentorship. By examining both PhD students and advisors, the research can foster constructive dialogue by revealing information about how students and advisors perceive each other’s goals for graduate student career development.

### The Pathway to the Professoriate: Choices and Context

The journey from an undergraduate major in STEM to the professoriate involves making difficult choices and investing energy in uncertain paths. PhD students may be guided in these choices by their academic advisors, who have achieved professorial positions. They may also be guided by cues they observe in their academic environment, cues that could affect their sense of belonging in academia (Walton and Cohen, 2007; Purdie-Vaughns et al., 2008) and their perceived social support in their department. Cues that signal belonging foster greater connection to an academic setting and shape an individual’s self-concept (Cohen and Garcia, 2008; Walton et al., 2012), and interventions that secure belonging in potentially threatening academic environments can lead to long term positive outcomes (see Walton and Brady, 2020 for review). For URM students^1^ and those who are the first of their family to attend college (hereafter first-generation students or FG), there may be additional uncertainty surrounding their graduate school experiences that may further impact their feelings of belonging in academia (Walton and Cohen, 2007; Byars-Winston, 2014; Mosley and Hargrove, 2014; Council of Graduate Schools, 2015).

The extent of URM and FG representation in the professoriate can impact a student’s desire to pursue and ability to complete a graduate degree, by framing their ability to imagine themselves succeeding in those roles (see Smith et al., 2002 for discussion). Approximately 12% of all full-time faculty in degree-granting postsecondary institutions are underrepresented minorities, while the remaining professoriate consists of 75% White and 11% Asian/Pacific Islander individuals (statistics as of 2018; National Center for Engineering and Statistics, 2020). Statistics on faculty members who identify as first-generation (FG) college students are less readily available. One national survey that contained data about faculty member’s parental education was conducted in 1999, revealing that FGs represented approximately 25% of all faculty members in R-1 (i.e., PhD granting universities with very high research activity) and R-2 universities (i.e., PhD granting universities with high research activity; National Center for Education Statistics, 2002).

Research using different methodologies from various disciplines conducted with students throughout the academic pipeline suggests that greater diversity in the professoriate may relate to differential educational and professional choices. Qualitative research with undergraduate students who are Latinx and FG revealed that those who had faculty mentors who could better relate to their cultural identities and provide guidance and insight about applying for graduate school expressed greater interest in pursuing doctoral study themselves (Martinez, 2018; see also Brazziel and Brazziel, 2001). Without a role model to provide adequate guidance in research and academia, many undergraduate URM and FG students may overlook their potential as scientists and the possibility of pursuing a graduate degree. At the graduate level, a good relationship between PhD students and their advisors is an important factor for thriving (see Brunsma et al., 2017 for review). PhD students who had positive perceptions of their relationships with their advisors met more frequently with their advisors (Heath, 2002), had a greater sense of belonging in their academic department (Lovitts, 2001), and were less likely to leave their doctoral studies before completion (Golde, 2005). Such positive relationships are particularly beneficial and crucial for students from underrepresented backgrounds, yet these groups of students may be at a disadvantage due to many faculty advisors’ lack of experience in mentoring them (Davis, 2008). Minority PhD students who perceived greater social support and sense of belonging viewed themselves as competent and successful (Ostrove et al., 2011), completed graduate school at higher and quicker rates (Lovitts, 2001; Curtin et al., 2013), and were more likely to pursue a research career after graduating (Spalter-Roth et al., 2013).

However, when faculty advisors are less aware of the challenges that URM and FG students face (e.g., lack of understanding of graduate education systems or lack of familial experience in higher education), they may fail to provide adequate instrumental and social support to address their students’ needs (Davidson and Foster-Johnson, 2001). Indeed, a recent study has identified the persistence of this issue. This study of 1,375 graduate students in the 100 chemistry departments in the United States that receive the greatest share of federal research funding found that women, and URM women in particular, reported fewer positive interactions with their faculty advisors. Moreover, URM students, and URM men in particular, reported receiving less than desired amounts of interpersonal support (Stockard et al., 2021).

Furthermore, some URM students express an inability to “fit the mold” of what is expected of them from their departments (Gardner, 2010a). Similar sentiments were shared among FG students who expressed that they do not “know the rules” of the system and that they are “living in two worlds,” needing to switch between identities as a family member and as a graduate student (Gardner and Holley, 2011). URM students and FG students are statistically more likely to come from lower socioeconomic backgrounds, which place greater emphasis on community and strong social ties, and thus, they may be less accustomed to the independent norms in academia (Stephens et al., 2012). Family members who are less familiar with academic norms may not provide the same knowledge and support, leading some students to be more attuned to the norms of faculty advisors. In addition to the challenges imposed by coursework and research, these sociocultural factors can exacerbate URM and FG students’ perceived lack of belonging and social support. For these reasons, interactions with advisors can powerfully influence decisions about what type of career to pursue after completing their doctorate.

### Norms and Conversations About Career Choices

Departmental norms and the specific relationships that PhD students have with their advisors have implications for PhD students’ career trajectories. In an academic research institution (i.e., at an R-1 university) for doctoral students, the injunctive norms (i.e., the perception of what most people approve or disapprove of) and the descriptive norms (i.e., the perception of what most people do) both support pursuit of an academic research career at an R-1 university (Golde, 2004, 2005). While PhD students often rely on their advisors for career advice, this may become challenging for students who are less interested in pursuing an academic career. A survey of doctoral students across life sciences, physics, and chemistry, revealed that students who are toward the end of their program (preparing for employment) rated a non-academic career as more attractive and a faculty career as less attractive compared to ratings of less advanced students (i.e., students who have not completed their qualifying exams; Sauermann and Roach, 2012). However, when asked about the type of careers encouraged by their advisors, students generally perceived a strong expectation that they pursue academic research positions. Moreover, little research has examined different types of academic positions, such as the research-oriented vs. teaching-oriented faculty positions. If students believe that they will no longer receive support for pursuing other career paths beyond academic research positions, they may opt to leave their graduate program before completion (Golde, 2005). Given the centrality of a faculty advisor in shaping their students’ future careers, conversations about career preferences are important.

While faculty advisors are the central resources for PhD students who wish to pursue academic careers, students with non-academic career goals often obtain their information from other sources. Interviews with 104 PhD students across 60 US chemistry departments revealed that PhD students lack awareness of specific career paths besides the two broad options of academia and industry and lack understanding of the skill sets and responsibilities required by non-academic positions (Thiry et al., 2015). With fewer resources outside of their programs (e.g., familial guidance, professional role models), URM and FGs, in particular, reported less awareness of other career options. When asked about the sources of their career information, students reported that they primarily learned from peers who were already in their job-search process. Only about 29% of the students mentioned that they learned about non-academic careers from their current advisor. Students who did not seek information from their advisors perceived their advisors to be unhelpful toward, and even openly unsupportive of, their decision to pursue a non-academic career (Thiry et al., 2015).

Similar patterns have been found in other studies examining faculty and program support for PhD students with non-academic career goals. Although PhD students expressed interests in career options besides tenure-track faculty positions, those with non-academic career goals perceived lower levels of support from their advisors and programs and were less likely to seek advice from their advisor or other faculty members (Golde, 2004; O’Meara et al., 2014; St. Clair et al., 2017). Faculty members may find it more difficult to provide advice on other career paths due to their own focus on academic research and lack of knowledge about other careers. The lack of role models for other career paths, along with the perceived lack of support from their academic advisors, contributes to students’ experience of low self-efficacy in their career advancement and a lack of perceived belonging in their program (O’Meara et al., 2014; Thiry et al., 2015; Jaeger et al., 2017).

Faculty advisors are aware of their role as a resource for graduate students for career advice, but some may overlook the possibility or not feel prepared to assist with their students’ career preferences if they are not in academia (Gardner, 2010b). Traditionally, the role of a faculty advisor has been to train the next generation of independent researchers for academic positions (Gardner, 2010b), and many students do enroll in doctoral programs with an aspiration to be a professor (Golde and Dore, 2001; Fuhrmann et al., 2011). However, as students progress in their doctoral programs, their interest in pursuing an academic career path often shifts (Fuhrmann et al., 2011; Sauermann and Roach, 2012). Without adequate communication, a mismatch between faculty advisors and PhD students can arise. For instance, with the assumption that their students are still interested in an academic research career, faculty advisors may not change their approach to career-related guidance, even if they have the resources and experience to advise them about non-academic positions. In turn, students may perceive their advisors as unhelpful in their non-academic career development and may be less likely to seek advice from them when faculty members may, as the opening quote illustrates, be quite willing to seek resources to assist them.

Although it is important to have conversations about career development, the prevalence or content of such conversations between PhD students and their advisors is unclear, despite the call in the sciences for PhD students and faculty advisors to create individual development plans (IDP; Austin and Alberts, 2012). Prior research suggests that these conversations do not typically occur until the student is already in the job-hunting process, if they happen at all (Golde, 2005; Fuhrmann et al., 2011; Haley et al., 2014). Bounded by the norms supporting the pursuit of a tenure-track academic position, students who wish to pursue a non-academic career may not be comfortable revealing their career preference to their advisors while they still need their support to complete the degree program. Such discomfort may be greater for URM and FG students, who may already question whether they fit in with expectations at their academic departments (Gardner, 2010a).

### Overview of Studies

The present research investigates potential gaps in career preferences and expectations between graduate students and advisors in STEM fields and the implications of possible discrepancies in normative perceptions. To foster better communication between PhD students and faculty advisors requires identifying what each group actually believes about different career options. Therefore, we conducted two studies to examine the perceptions that PhD STEM students and STEM faculty advisors have of different career paths and the desired options students have for themselves (Study 1) and advisors have for students they advise (Study 2). This research examines academic vs. non-academic options (as previous research has done) and looks at different types of career paths (research vs. teaching) within academia.

More specifically, in Study 1, a survey of STEM PhD students at two R-1 universities, we examine the following questions:

1. Do PhD students prefer a career in research-focused positions, teaching-focused positions, or non-academic positions?
2. Out of those three career options, what do PhD students believe their faculty advisors prefer, both in general (i.e., their normative perceptions) and for them in particular?
3. How does PhD student career preference relate to their sense of belonging and perceived social support?
4. How comfortable are PhD students in discussing these different career options?
5. Are the patterns of career preferences identified similar or different for students as a function of their status as first (vs. continuing) generation students or as underrepresented minorities vs. non-underrepresented minorities? We include these analyses to examine whether interest in different careers, and perceptions of belonging and support, would be different for those from these groups that are traditionally less represented in PhD programs.

In Study 2, a survey of STEM faculty advisors in the same two R-1 universities, we examine the parallel questions about how they think about advising students in general, and specific students they are currently advising in particular. (The two studies are independent, and thus the advisors from Study 2 were not matched, or able to be matched, with the particular students from Study 1). To facilitate better dialogue between PhD students and advisors requires understanding the perceptions that each side has of the other, as well as their meta-perceptions (i.e., what do PhD students think that their advisors are thinking about them.) Together, the goal of these studies is to paint a portrait of how the experience and preferences of PhD students and faculty advisors are shaped by their perceptions of the career norms and their expectations of each other’s preferences. By examining perceptions of *both* sides and their responses to analogous questions about each other, there is a greater opportunity for identifying inaccurate perceptions of norms where they exist.

Data, code, materials, and supplemental analyses for both studies are available at https://osf.io/4uyxh/. Data analyses were not conducted until data collection was complete.

## Study 1

### Method

#### Participants

One hundred ninety-five PhD students from the University of California Santa Barbara (UCSB, *N* = 123) and the University of California Merced (UCM, *N* = 72) completed an online survey after being recruited *via* email. Students were recruited from all STEM disciplines as defined by the National Science Foundation (NSF). We also used the NSF categorization of Blacks, Hispanics, and Native Americans/Native Alaskans as being from under-represented groups in science and engineering professions (National Science Foundation, 2017). 35.9% of participants in the sample were students from under-represented racial/ethnic groups. 33.3% of participants were FG college students (FG). 57.4% had advanced to candidacy, 41.5% had not, and 1.0% did not report their candidacy status. All participants were compensated with a $10 electronic gift card. Table 1 lists complete demographics, including the discipline of study (see Supplementary Material for additional information about PhD student sample and population characteristics).

**Table 1:** Students’ demographic characteristics.

Characteristics		*N*(%)	*N*(%)
Age *M*(*SD*)	27.81 (3.51)	
School		
UC – Santa Barbara		123 (63.1)
UC – Merced		72 (36.9)
Gender		
Male		90 (46.2)
Female		102 (52.3)
Other/Missing		3 (1.5)
Race		
Asian/Asian-American		24 (12.3)
Black/African-American		17 (8.7)
Hispanic/Latino-American		46 (23.6)
Native American		4 (2.1)
Native Pacific Islander		3 (1.5)
Other/Missing		14 (7.2)
White/Caucasian American		87 (44.6)
URM status		
URM		70 (35.9)
Non-URM		123 (63.1)
Other/Missing		2 (1.0)
Year in PhD program		
First-year		3 (1.5)
Second-year		57 (29.2)
Third-year		44 (22.6)
Fourth-year		34 (17.4)
Fifth-year		38 (19.5)
Sixth-year		16 (8.2)
Seventh-year or more		3 (1.5)
Advancement status		
Pre-advancement		112 (57.4)
PhD candidates		81 (41.5)
Other/Missing		2 (1.0)
National status		
International student		45 (23.1)
Domestic student		149 (76.4)	Other/Missing		1 (0.5)
College generation status		
First-generation college student		65 (33.3)
Continuing-generation college student		129 (66.2)
Other/Missing		1 (0.5)
Field of study		
Engineering		39 (20.0)
Life and environmental sciences		45 (23.1)
Other/Missing		11 (5.6)
Physical sciences		33 (16.9)
Social sciences		67 (34.4)

#### Procedure

A sample of 500 graduate students from UCSB (*N* = 350) and UCM (*N* = 150) in STEM disciplines was recruited to complete the survey online using their university email addresses.^2^ Participants were contacted by the graduate divisions of their respective universities. Students from the NSF-defined URM groups were over-sampled based on demographic information obtained by the graduate division in order to ensure sufficient representation for analyses. The 195 students who responded and completed the study corresponds to a 39% completion rate (UCM = 47%, UCSB = 35%). Ethics approval was granted by the Human Subjects Committee at the University of California, Santa Barbara. Informed consent was given digitally at the beginning of the survey before proceeding to the following measures.

### Measures

#### Desirability of Career Options

Participants were first asked to assess the desirability of three different career options after the completion of their PhD. These options were divided into broad categories and always presented using the same terminology: “non-academic position (e.g., industry, government, non-profit organization),” “teaching-focused academic position (professor at college *without* a PhD program),” and “research-focused academic position (professor at university *with* a PhD program).”

Participants indicated the desirability of these three options in two ways – one that resulted in a categorical variable and one that resulted in a continuous variable. First, they ranked the three options based on *their personal preferences* such that 1 indicated the option most desirable to them, 2 indicated an option moderately desirable to them, and 3 indicated the option least desirable to them. This forced choice was intended to categorize their priorities. Second, they rated the desirability of each of the three options on a scale from 0 (not at all desirable) to 10 (extremely desirable).

Participants then completed the same ranking and desirability questions for the same three career options but this time from *their advisor’s perspective*, as they understood it. Participants reported what they believe their advisor would prefer for them personally (i.e., the PhD student) to pursue professionally after completing their degree. Participants also answered the same set of questions about their perception of their advisors’ *general* preference for careers chosen by the various PhD students they train.

To assess department norms, the participants also completed the same ranking and desirability questions about the perceived preferences of *other faculty members* in their department *in general,* as well as their perception of how *other graduate students* in their department were thinking about their specific careers. Thus, in total, participants indicated their ranking and scaled desirability of the same three career options from their perspective, their perception of their advisors’ preference *for them*, their perception of their advisors’ preference *in general*, their perception of other faculty members’ preferences *in general*, and their perception of other graduate students’ preferences for *their own* specific careers.

#### Comfort With Discussion

Participants reported how comfortable they felt discussing each of the three career options with their advisor and other faculty members in the department. Participants responded on a scale from 0 (very uncomfortable) to 10 (very comfortable). They were also provided with space to elaborate on their reasons for feeling comfortable or uncomfortable with these conversations.

#### Perceived Social Support

Participants completed an adapted version of a perceived social support scale (Zimet et al., 1988) which was designed to assess their experience of feeling valued, cared about, and respected by important others. Modifications to the 8-item scale were made to focus on the experience of feeling socially supported by their PhD advisors specifically; responses ranged from 1 (strongly disagree) to 7 (strongly agree; *M* = 5.52, *SD* = 1.23, *α* = 0.93). Sample items include, “My graduate advisor is available when I need to meet,” and “I can count on my graduate advisor when things are not going well.”

#### Belonging

Participants completed an adapted version of the Belonging Scale (Walton and Cohen, 2007), which was designed to assess the extent to which people feel as though they are liked and accepted within a particular context (Walton and Cohen, 2007). Participants reported their feelings of belonging within their academic department on 11 items; responses ranged from 1 (strongly disagree) to 7 (strongly agree; *M* = 4.73, *SD* = 1.20, *α* = 0.92). Sample items include, “I fit in well in my academic department,” and “I am similar to the kind of people who succeed in my academic department” and “When something bad happens, I feel that maybe I don’t belong in my academic department (reverse scored).”

#### Additional Measures

Several additional measures were included that focused on views of faculty advisors and different professional development opportunities (included in Supplementary Material in Open Science Framework).

### Results

#### PhD Students’ Normative Perceptions of Career Preferences in Their Academic Departments

To determine how PhD students perceived the norms in their academic departments, we assessed their perception of their advisors’ general career preferences, their perception of other faculty members’ general career preferences, and their perception of other PhD students’ career preferences. We assessed these perceptions in two ways, categorical rankings and numerical ratings, and the results were generally consistent for the two types of measures. We examined the three occupational categories the PhD students ranked highest on the 1–3 ranking scale to determine their categorical preferences. Table 2, first row, indicates that PhD students’ perceptions of advisors’ general preferences strongly supported research careers, with 81.1% ranking that option highest, 13.0% ranking non-academic careers highest, and 5.9% ranking teaching highest. Other faculty in the academic department were also viewed as primarily supporting research careers, as shown in Table 2, second row. Thus, the norms of the department faculty across STEM fields are seen as being strongly in favor of research as perceived by PhD students in their programs.

**Table 2:** Frequency table for PhD student’s self and perceived others’ career preferences.

	Non-academic	Teaching	Research	Total
	*N*(%)*	*N*(%)	*N*(%)	*N*
Normative perceptions				
Perception of advisors’ general career preference	22 (13.0)	10 (5.9)	137 (81.1)	169
Perception of other faculty members’ general career preference	16 (9.3)	3 (1.7)	153 (89)	172
Perception of other PhD students’ career preference	81 (45.5)	20 (11.2)	77 (43.3)	178
Personal perceptions				
PhD students’ self-reported career preferences	89 (45.6)	40 (20.5)	66 (33.8)	195
PhD students’ perception of advisors’ career preference for them	23 (13.9)	12 (7.2)	131 (78.9)	166

* Note that % excludes missing cases.

Participants saw their fellow PhD students (Table 2, third row), by contrast, as being much more balanced in their career preferences: 43.3% ranked research highest, 45.5% ranked non-academic highest, and 11.2% ranked teaching highest. In sum, PhD student participants saw a divide between faculty preferences for students in general and the preferences of their fellow students for their careers. They perceived the normative faculty preference as oriented almost solely toward research whereas the normative PhD student preference was more balanced between research, teaching, and industry positions.

**Figure 1:**
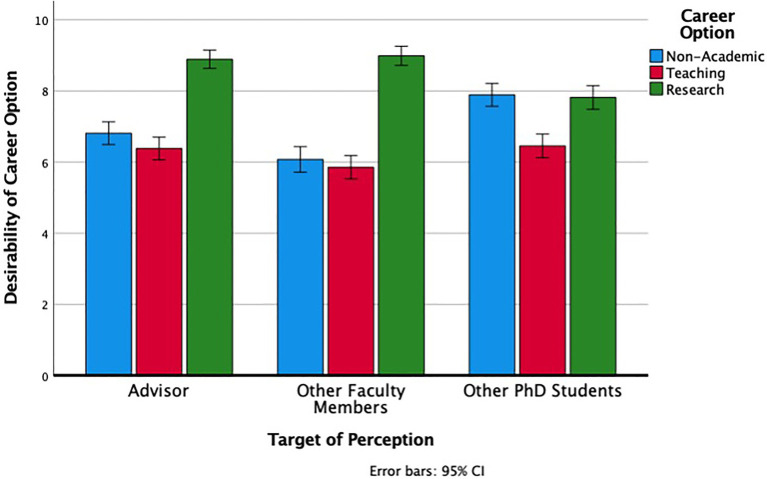
PhD students’ perceptions of advisors’ general desirability ratings, faculty members’ general desirability ratings, and other graduate students’ desirability ratings for their own careers for each career option.

Participants’ continuous assessments of the perceived desirability of the different career options (on scales from 0 to 10) were consistent with their rankings. We conducted a 3 (Career Option: Non-Academic, Teaching, Research) × 3 (Target of Perception: Advisors, Other Faculty Members, Other PhD Students) Repeated Measures ANOVA. There was a main effect of career option, *F* (2, 384) = 15.9, *p* < 0.001, *η_p_^2^* = 0.08, a main effect of target of perception, *F* (2, 384) = 88.13, *p* < 0.001, *η_p_^2^* = 0.32 and an interaction between the two, *F* (4, 768) = 115.77, *p* < 0.001, *η_p_^2^* = 0.17. The nature of the interaction (see Figure 1) was that for estimates of general faculty preferences (both other faculty in the department and students’ own advisors), research careers were the most desirable with teaching and non-academic options roughly similar. By contrast, students perceived their fellow PhD students as seeing both non-academic and research careers being more desirable, and teaching less so. In short, across both categorical and continuous measures, there was a discrepancy between what students perceived as the normative career preferences among faculty in general and among their fellow PhD students.

#### PhD Students’ Own Career Preferences, and What They Perceive Their Advisors’ Preferences Are for Them

We next examined how PhD students view those same three career options for themselves specifically and how they see their advisors’ preference for them as students in particular. We examined the three occupational categories the PhD students ranked highest on the 1–3 ranking scale to determine their categorical preferences. Table 2, fourth row, indicates that 45.6% of PhD students ranked non-academic careers highest, 20.5% ranked teaching careers highest, and 33.8% ranked research careers highest. This contrasted with what the students perceived as their advisors’ preferences for them. Table 2, fifth row, indicates that the vast majority of students, 78.9%, perceived that their advisor would rank research careers highest, whereas 13.9% thought their advisor would rank non-academic careers highest, and 7.2% thought their advisor would rank teaching career highest. In short, the PhD students perceived that their advisors wanted them to go into research – that their advisors’ preferred career choices for them, in particular, were similar to their advisors’ general career preferences. This was discrepant from their own preferences, that were much more balanced across the options.

**Figure 2:**
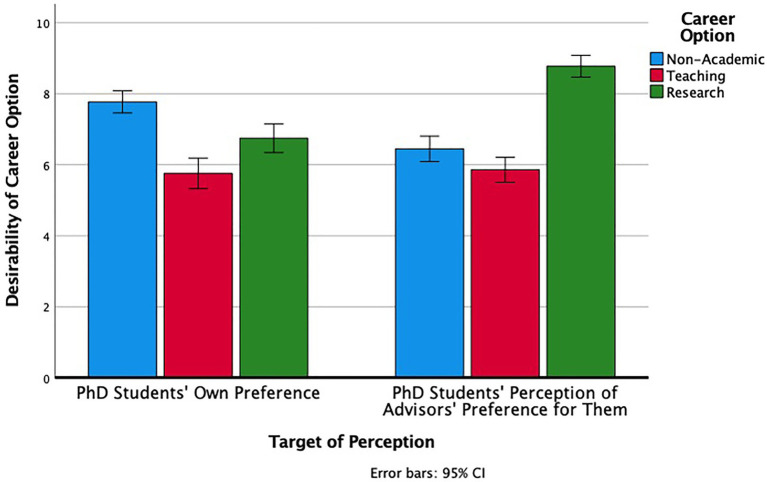
PhD students’ desirability ratings for each career option (left), and their perception of their advisors’ desirability ratings for each career option for them (right).

This discrepancy was represented in PhD students’ continuous perceptions as well. We conducted a 3 (Career Option: Non-Academic, Teaching, Research) × 2 (Target of Perception: PhD Students’ Own Preference, PhD Students’ Perception of Advisors’ Preference for Them) Repeated Measures ANOVA. There was a main effect of career option (Non-Academic: *M* = 7.11, *SD* = 1.90; Teaching: *M* = 5.81, *SD* = 2.29; Research: *M* = 7.76, *SD* = 1.98), *F* (2, 388) = 41.31, *p* < 0.001, *η_p_^2^* = 0.18. There was also a main effect of target (PhD Students’ Own Preference: *M* = 6.76, *SD* = 1.27; PhD Students’ Perception of Advisors’ Preference for Them: *M* = 7.03, *SD* = 1.33), *F* (1, 194) = 6.73, *p* = 0.01, *η_p_^2^* = 0.03, and an interaction between the two, *F* (2, 388) = 49.93, *p* < 0.001, *η_p_^2^* = 0.21. As Figure 2 illustrates, PhD students saw their advisors as strongly preferring research careers for them over the other two options, whereas they preferred non-academic positions most and were much more balanced, overall, in their assessments of the three options. Thus, PhD students as a whole perceived a discrepancy between what they wanted for their post-PhD career and what their advisors wanted for them. We turn next to examining demographic and categorical differences in career preferences to identify similarities and differences across categories associated with greater (vs. lesser) representation in PhD programs.

#### Demographic and Categorical Differences in Preferences for Career Options

Next, we examined whether the categorical preferences varied as a function of the participants’ URM and FG status. A series of *χ*^2^ analyses in Table 3 indicates that across URM-status and FG status, participants were balanced in their preferences for research and non-academic positions, with teaching positions being clearly less preferred whereas they perceived that their advisors preferred them to go into research. That is, the trends observed overall for PhD students were consistent across URM status and generation status.

**Table 3:** PhD students’ own and perception of advisors’ preferences for them by demographic categories.

	Non-academic	Teaching	Research	Total	*χ* ^2^
	*N*(%)	*N*(%)	*N*(%)	*N*	
PhD students’ preferences					
URM	32 (45.7)	14 (20 .0)	24 (34.3)	70	*χ*^2^ (2, *N* = 193) = 0.003,*p* = 0.99
Non-URM	56 (45.5)	25 (20.3)	42 (34.1)	123	
PhD students’ perception of advisors’ preferences for them					
URM	6 (10.5)	6 (10.5)	45 (78.9)	57	*χ*^2^(2,*N* = 166) = 2,*p* = 0.37
Non-URM	17 (15.6)	6 (5.5)	86 (78.9)	109	
PhD students’ preferences					
FG	26 (40 .0)	17 (26.2)	22 (33.8)	65	*χ*^2^(2, *N* = 194) = 2.52, *p* = 0.29
CG	63 (48.8)	22 (17.1)	44 (34.1)	129	
PhD students’ perception of advisors’ preferences for them					
FG	7 (12.7)	7 (12.7)	41 (74.5)	55	*χ*^2^ (2, *N* = 166) = 3.71, *p* = 0.16
CG	16 (14.4)	5 (4.5)	90 (81.1)	111	

A similar conclusion was obtained when we examined continuous assessments of the same variables – PhD students’ career preferences – as a function of participants’ URM and FG status. We conducted a 3 (Career Option: Non-Academic, Teaching, Research) × 2 (URM status: URM, non-URM) mixed-model ANOVA. There was a main effect of career option (as noted above). There was no main effect of URM status (URM: *M* = 6.81, *SD* = 2.11; non-URM: *M* = 6.77, *SD* = 1.59), *F* (1, 191) = 0.06, *p* = 0.80, *η_p_^2^* < 0.001. Critically, there was no interaction between the two factors, *F* (2, 382) = 0.29, *p* = 0.75, *η_p_^2^* = 0.01. The PhD students rated research and non-academic positions as more desirable than teaching positions, and this was consistent across URM status. A similar mixed-model ANOVA with participants’ generation status as the between-subject variable revealed a consistent pattern. Again, there was a main effect of career option. There was no main effect of first generation (FG) status (FG: *M* = 6.74, *SD* = 2.21; Continuing Generation (CG): *M* = 6.78, *SD* = 1.56), *F* (1, 192) = 0.04, *p* = 0.84, *η_p_^2^* < 0.001. And critically, there was also no interaction between the two factors, *F* (2, 384) = 0.49, *p* = 0.62, *η_p_^2^* = 0.003. FGs and CGs rated research and non-academic positions as more desirable than teaching positions. In all, our results revealed no demographic differences in career interest.

Moreover, neither URM status nor generation status moderated these assessments when examined continuously. We conducted a 3 (career option: non-academic, teaching, research) × 2 (URM status: URM, non-URM) mixed model ANOVA with perceived advisor’s desirability for them to pursue each career option as a dependent variable. There was a significant main effect of career option (as noted above), and no main effect of URM status (URM: *M* = 6.88, *SD* = 2.18; non-URM: *M* = 7.14, *SD* = 1.65), *F* (1, 191) = 1.74, *p* = 0.19, *η_p_^2^* = 0.01. There was no significant interaction, *F* (2, 382) = 1.42, *p* = 0.24, *η_p_^2^* = 0.01 as both URMs and non-URMs perceived that their advisor had a strong desirability for them to pursue research positions compared to non-academic and teaching positions. For generation status, there was a main effect of FG status (FG: *M* = 6.75, *SD* = 2.28; CG: *M* = 7.17, *SD* = 1.62), *F* (1, 192) = 4.24, *p* = 0.04, *η_p_^2^* = 0.02, but no interaction between career option and generation status, *F* (2, 384) = 0.91, *p* = 0.40, *η_p_^2^* = 0.01. Taken together, there was consensus among all PhD students that their advisors perceived research careers to be most desirable, relative to the other options.

#### Relationship Between Career Preferences of PhD Students and Belonging and Perceived Social Support

**Figure 3:**
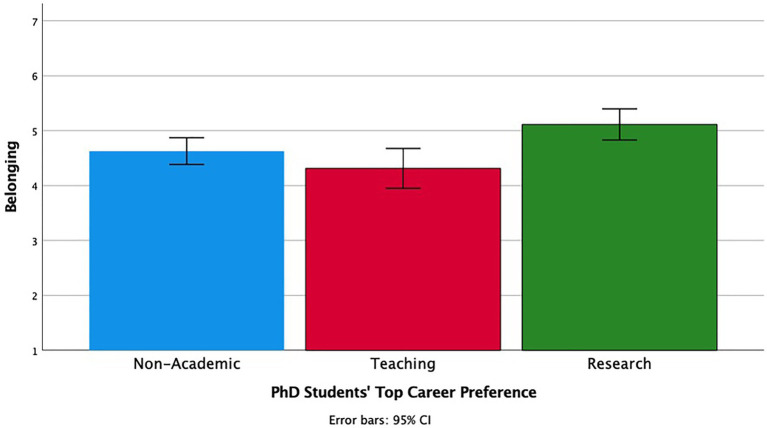
The relationship between PhD students’ own career preference and their sense of belonging in the academic department.

We next investigated the relationship of different career preferences to students’ feelings of belonging and perceived social support to answer the question as to whether students who ranked the normative choice (among faculty) as their highest choice feel the most belonging and supported. Students were classified based on their top-ranked career preference. We first conducted a Multivariate ANOVA (MANOVA) with belonging and social support as the outcomes and top-ranked career preferences (Non-Academic, Teaching, Research) as the independent variable. The main effect of career preference was significant on belonging, *F* (2, 192) = 6.48, *p* = 0.002, *η_p_^2^* = 0.06 (see Figure 3). Students who ranked research positions highest felt the most belonging (*M* = 5.11, *SD* = 1.20), followed by those who ranked non-academic positions highest (*M* = 4.63, *SD* = 1.16). Students who ranked teaching positions highest reported the least belonging (*M* = 4.31, *SD* = 1.14).

**Figure 4:**
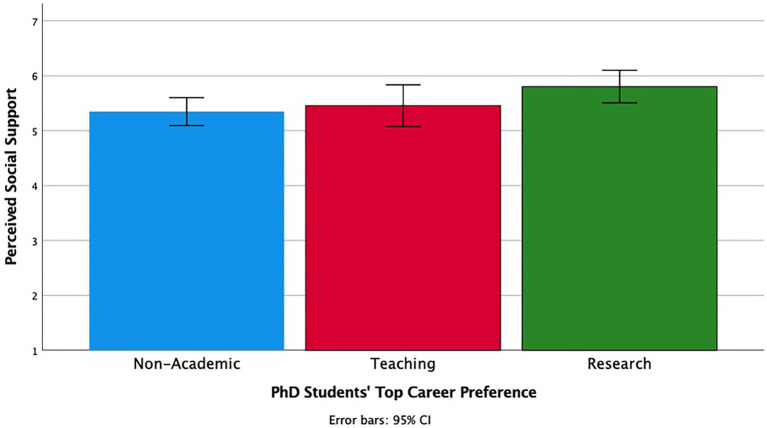
The relationship between PhD students’ own career preference and perceived social support within the department.

There was also a marginally significant effect of career preference on perceived social support, *F* (2, 192) = 2.75, *p* = 0.067, *η_p_^2^* = 0.03 (see Figure 4). Students who ranked research positions highest perceived the greatest social support from their advisors (*M* = 5.80, *SD* = 1.13), followed by students who ranked teaching positions highest (*M* = 5.46, *SD* = 1.12). Students who ranked non-academic positions highest perceived the least amount of social support (*M* = 5.35, *SD* = 1.32). In short, PhD students whose career preferences were consistent with what was normative among faculty felt the most belonging in the department and felt most socially supported by their advisors.

#### Demographic Differences in the Impact of Career Preferences on Belonging and Support

Next, we conducted a series of analyses to examine whether this greater feeling of belonging among those who preferred research was similar or different as a function of URM and college generation status. The results, in short, indicated consistency in findings across both variables and important main effects as a function of the demographic variables. First, we conducted a 3 (Career Preference: Non-Academic, Teaching, Research) × 2 (URM status: URM, non-URM) between-subjects ANOVA with belonging as the dependent variable. There was a main effect of career preference (as noted above) and a main effect of URM status (URM: *M* = 4.25, *SD* = 2.01; non-URM: *M* = 4.94, *SD* = 1.51), *F* (1, 187) = 14.85, *p* < 0.001, *η_p_^2^* = 0.07. However, there was no interaction between the two factors, *F* (2, 187) = 1.18, *p* = 0.31, *η_p_^2^* = 0.01. Similarly, while there was a significant main effect of generation status (FG: *M* = 4.29, *SD* = 2.00; CG: *M* = 4.90, *SD* = 1.54), *F* (1, 188) = 11.65, *p* = 0.001, *η_p_^2^* = 0.06, there was no significant interaction between the two factors *F* (2, 188) = 0.23, *p* = 0.80, *η_p_^2^* = 0.01. FGs felt less belonging than CGs, and URM students felt less belonging in their academic departments than non-URM students. However, these factors did not moderate the relationship between career choice and belonging in the department.

We ran a similar analysis examining the impact of URM status, college generation status, and career preference on how graduate students felt socially supported by their advisors. There was no main effect of URM status, *F* (1, 187) = 1.63, *p* = 0.20, *η_p_^2^* = 0.01 and no interaction between URM status and career choice, *F* (1, 187) = 0.72, *p* = 0.49, *η_p_^2^* = 0.01. URM students felt equally supported by their advisors as non-URM students, regardless of their career preferences. Similarly, there was no main effect of college generation status, *F* (1, 188) = 1.50, *p* = 0.22, *η_p_^2^* = 0.008 and no interaction with career choice, *F* (2, 188) = 0.08, *p* = 0.93, *η_p_^2^* = 0.001. FGs felt equally supported by their advisors as CGs, and URMs felt equally supported as non-URMs, regardless of their career preferences.

#### Comfort in Discussing Different Career Options

We investigated students’ comfort in discussing different career options with their advisors. Would students feel more comfortable discussing what they perceived to be the more preferred option among their faculty advisors? We first conducted a repeated-measures ANOVA, with comfort discussing the three career options (Non-Academic, Teaching, and Research) as the within-subject variable. PhD students were much more comfortable discussing research careers, the career option that they perceived as normative among the faculty. The repeated measures ANOVA revealed a significant difference, *F* (2, 388) = 44.87, *p* < 0.001, *η_p_^2^* = 0.19. Students felt more comfortable discussing research positions with their advisors (*M* = 8.82, *SD* = 1.99) than non-academic positions (*M* = 7.32, *SD* = 2.95), *p* < 0.001 or teaching-focused positions (*M* = 7.23, *SD* = 2.72), *p* < 0.001, with no difference between teaching and non-academic, *p* = 0.57.

Next, we tested whether the URM status or generation status of graduate students affects their comfort level in discussing different career options with their advisors. We conducted a 3 (Career Option: Non-Academic, Teaching, Research) × 2 (URM Status: URM, non-URM) Mixed Model ANOVA predicting students’ comfort in discussing each career option. There was a main effect of different career options (as noted above), and a main effect of URM status (URM: *M* = 7.38, *SD* = 3.46; non-URM: *M* = 8.03, *SD* = 2.61), *F* (1, 191) = 4.39, *p* = 0.04, *η_p_^2^* = 0.02. Overall, non-URM students were more comfortable discussing different career options with their advisors compared to URM students. There was no interaction between the two factors, *F* (2, 382) = 2.14, *p* = 0.12, *η_p_^2^* = 0.01. Conducting analogous analysis with generation status revealed no main effect of generation status, *F* (1, 192) = 1.90, *p* = 0.17, *η_p_^2^* = 0.01, and no interaction between the two factors, *F* (2, 384) = 1.47, *p* = 0.23, *η_p_^2^* = 0.01. Overall, all PhD students were more comfortable discussing careers related to research with their advisors, compared to teaching and non-academic positions.

### Discussion

In Study 1, we first examined whether there was a discrepancy in normative career preferences of PhD students and what they perceived to be their faculty advisors’ career preference for them. We were particularly interested in whether these patterns differ as a function of students’ URM or generation status. Using both categorical and continuous measures, we found that PhD students, regardless of their demographic backgrounds, preferred non-academic positions and research positions roughly equally, followed by teaching positions, and that they perceive a similar distribution among their peers. However, students perceived their advisors and other faculty members in their department as strongly preferring research positions with no difference in preference between teaching and non-academic positions. In general, there were discrepancies between students’ career preferences and the careers that they thought their advisors wanted them to pursue after graduation. We speculate that this resulted in several important consequences for the student-advisor relationship.

First, this discrepancy may have contributed to a lower sense of belonging in their department and perceived social support from their advisors. In general, students who preferred research positions felt the most belonging and social support. An important caveat to note is that these factors almost certainly vary as a function of the year in the program and academic discipline – for example, second- and sixth-year students and psychology and engineering students were all included in the sample and likely differ meaningfully. However, the sample size did not enable a detailed examination at that level. We chose, rather, to focus on two moderators related to diversity in the professoriate – URM status and generation status.

In considering students’ demographic backgrounds, URM and FG students felt less belonging yet equally supported overall relative to non-URM and continuing generation students. The lack of interaction suggested that coming from different demographic backgrounds and having distinct career preferences did not exacerbate or bolster students’ sense of belonging and perceived social support.

Second, students’ comfort in having career discussions with their advisors differed depending on the career options. Students were most comfortable discussing research positions, the positions they perceived as most normatively preferred among faculty at their departments. We also found an effect of students’ URM status. Overall, URMs were less comfortable having career discussions with their advisors than non-URMs; there were no differences between FGs and CGs.

Taken together, Study 1 provides a clearer picture of the discrepancies between what PhD students perceive as the norm in their departments and what they desire for their own careers. Students see their advisors as not being particularly attuned to their own interests. Although many students desire teaching or non-academic careers, they do not feel as comfortable discussing these careers with their advisors as they do for discussing research careers.

URM and FG students overall experienced less perceived belonging in their department, although this did not interact with their career choices. Regardless of whether they desired to pursue research, teaching, or industry, URM and FG students felt less belonging, and URM students felt less supported. This may be related to their hesitation to discuss these different career paths.

To put these results into a fuller context, it is important to examine the perspective of faculty advisors who are mentoring PhD students in STEM fields. We turn to that in Study 2.

## Study 2

In Study 1, PhD students in STEM fields perceived that their advisors strongly favored academic research careers for them, whereas they were more evenly divided in what careers they desired most for themselves among non-academic and academic research careers, and to a lesser extent, academic teaching careers. To the extent that actions are driven by perceptions of norms, it is important to determine, broadly speaking, the accuracy of these norms.

We reasoned that faculty members might have competing motivations for the career preferences of their students. As indicated with the quote from a faculty member to begin the paper, they may prefer entering PhD students to pursue academic research careers in the abstract. However, they may also see themselves as being responsive to the preferences of their specific advisees. Thus, faculty members may have discrepancies between what they prefer in general and what they prefer for particular students. Moreover, because discussions about different career options may be relatively rare (Fuhrmann et al., 2011), this flexibility may not be communicated in full to the PhD students. If the perception of PhD students in Study 1 is based on the view that advisors have in the abstract, and advisors feel differently in the abstract than they do about particular students they advise (as examined in Study 2), there may be room for both parties to communicate their goals more clearly and effectively.

We seek to further understand these issues in Study 2, conducted with a sample of STEM faculty advisors. We raise the following questions:

1. Out of the three career options, research-focused positions, teaching-focused positions, or non-academic positions, what do faculty advisors actually prefer, both in general and for specific students they are advising?
2. Are faculty advisors’ career preferences for students they advise more strongly related to their perceptions of the students’ preferences or their own general preferences?
3. How comfortable are faculty advisors having discussions related to each of the three career options?
4. How do faculty members perceive levels of belonging among students they advise, and how do these perceptions vary by their perceptions of characteristics of the students (their career preferences and demographics)?

By addressing these questions, our goal is to shed further light on the relationship between graduate students and their advisors. We note again that while the faculty members in Study 2 were from the same universities and departments as the PhD students in Study 1, they were not matched as the advisors of the student participants in Study 1, as each group responded voluntarily (and anonymously) to participate in the respective studies.

### Method

#### Participants

Three hundred one STEM faculty members from the University of California Santa Barbara (UCSB, *N* = 177) and the University of California Merced (UCM, *N* = 97) completed an online survey sent *via* email to all STEM faculty at the two universities (27 provided data and were included in analyses but did not include demographic characteristics including university). Our target sample was 300 to allow adequate coverage of the different disciplines, and we sent multiple contact emails in order to attain that. STEM disciplines were defined using the National Science Foundation standards. All participants were compensated with a $20 electronic gift card. Demographics of the sample are presented in Table 4 (see Supplementary Material for additional information about faculty sample and population characteristics).

**Table 4:** Faculty demographic characteristics.

Characteristics		N(%)
Age M(SD)	49.1(11.6)	
Years in Professoriate	16.2(12.1)	
School / Field of Study		
UC - Santa Barbara		177(64.6)
Social Sciences		52(29.3)
Life Sciences		48(27.1)
Physical Sciences		17(9.6)
Engineering		28(15.8)
Math		18 (10.2)
Other/Unspecified		14(7.9)
UC - Merced		97(35.4)
Social Sciences		33(34.0)
Life Sciences		18(18.5)
Physical Sciences		17(17.5)
Engineering		21(21.6)
Other/Unspecified		8(8.2)
Missing		27
Gender		
Male		165(60.7)
Female		105(38.6)
Other		2(0.7)
Missing		29
Race		
Asian / Asian-American		31(11.7)
Black / African-American		3(1.1)
Hispanic / Latino-American		21(7.9)
Multi-Racial		10(3.2)
Native American		1(0.4)
Other		12(3.8)
White / Caucasian American		188(70.7)
Missing		35
Professor Status		
Assistant Professor		75(27.4)
Associate Professor		51(18.6)
Full Professor		144(52.6)
Other		4(1.5)
Missing		27
US Born		
U.S. Born		176(64.9)
Non-U.S. Born		95(35.1)
Missing		30
College Generation Status		
First-Generation College Student		61(22.6)
Continuing-Generation College Student		209(77.4)
Missing		31

Note that % excludes missing cases

#### Procedure

A sample of 692 faculty members from UCSB (*N* = 525) and UCM (*N* = 167) in all STEM disciplines was recruited to complete the survey online using their university email addresses. All faculty members across all STEM disciplines at UCSB and UCM were recruited to complete the survey online using their university email addresses. The 301 faculty members who responded and completed the study corresponds to a 43.4% completion rate.^3^ Informed consent was given digitally at the beginning of the survey. Faculty were asked how many graduate students they were currently advising before proceeding to the dependent measures and were asked to complete all of the following measures for their three most senior current students (or fewer if they were currently advising less than three students). Faculty were informed that this was a study supported by the Graduate Divisions of both schools and funded by the National Science Foundation.

##### Desirability of Career Options

Faculty members were asked to assess the desirability of the same three career options that PhD students were asked to consider in Study 1, using the same language: “non-academic position (e.g., industry, government, non-profit organization),” “teaching-focused academic position (professor at college *without* a PhD program),” and “research-focused academic position (professor at university *with* a PhD program).” They ranked the three options based on *their personal preferences* such that 1 indicated the option most desirable to them, 2 indicated an option moderately desirable to them, and 3 indicated the option least desirable to them. This forced choice was intended to categorize their priorities.

First, faculty advisors ranked their *general preferences* for PhD students they may train. In particular, they were told: “We are interested in your general preferences for career options for the PhD students that you train. Please answer the next set of questions thinking about an ideal PhD student you recruit in the future.” Participants responded such that 1 indicated the option most desirable to them, 2 indicated an option moderately desirable to them, and 3 indicated the option least desirable to them. As in Study 1, this forced choice enabled a categorical assessment of their most highly preferred option. Second, they provided continuous assessments as they rated how desirable each of the three options was on a scale from 0 (not at all desirable) to 10 (extremely desirable). Thus, faculty advisors indicated the desirability of these three options in the same ways graduate students were asked about in Study 1.

Next, faculty advisors were asked to think of the three most senior students in their lab, labeling the most senior “Student A,” the second most senior “Student B,” and the third most senior “Student C.” The participants were then instructed: “For each student, we will first ask about your own perspective on his/her ideal career path and then ask what you believe that student’s preferences to be. Let us start with the most senior PhD student in your lab. We will call this person ‘Student A.’ If you have two equally senior students, choose either one as Student A. Please take a moment to bring an image of this student to mind and answer the next set of questions about him/her specifically.” After thinking about Student A, participants were asked: “In terms of career options for Student A after obtaining a PhD, please drag and drop the following options to rank them according to your own perspective” and used the same scale such that 1 indicated the option most desirable to them, 2 indicated an option moderately desirable to them, and 3 indicated an option least desirable to them. Participants then rated each option on a scale from 0 (not at all desirable) to 10 (extremely desirable).

Faculty participants then reported *their perception* of what each students’ preferred career path might be, as they understood it. Specifically, they ranked the same three options from Student A’s perspective, as they understood it from 1 (most desirable), 2 (moderately desirable), and 3 (least desirable) when Student A is thinking about his/her career options after obtaining a PhD. Participants then rated each option on the scale from 0 (not at all desirable) to 10 (extremely desirable).

Faculty participants then completed a shortened, 3-item belonging measure for each student, adapted from the scale used in Study 1 (Walton and Cohen, 2007), to assess how well they believed that student fit into the department. They made ratings on seven-point scales anchored at 1 (strongly disagree) and 7 (strongly agree) for Student A: “This student is similar to the kind of people who succeed in the department.” “This student gets along well with people in the department.” And “This student fits in well in the department.” The reliabilities were high for all students (*α* for belonging for Student A, B, C were 0.83, 0.84, 0.85, respectively). Finally, participants responded to the question: “Have you discussed career goals with Student A?” on a scale 1 (not at all) to 5 (a great deal). After completing these responses for Student A, participants evaluated Student B and Student C (their next most senior students, as applicable).

Faculty participants provided demographic details for each student they reported, including a year in the program, race, generation status, and gender identity.

In addition to the primary dependent measures, several additional measures (both quantitative and open-ended) were included to assess faculty members’ general preferences and perceptions, independent of any particular student (see Supplementary Material).

Finally, faculty participants indicated their own demographic information. Participants were then debriefed and thanked for their assistance.

### Results

#### Preferences for PhD Students’ Careers

We first examined the career that each faculty advisor would choose for a hypothetical “ideal” student they may imagine themselves working with, what we will refer to as *Advisor’s General Career Preference for PhD Students*. As can be seen in Table 5, top line, 84.0% of STEM faculty surveyed imagine their ideal student pursuing a research career, while 11.1% prefer a student who would pursue a non-academic career, and 4.9% prefer a student who would prefer a teaching-focused academic career.

**Table 5:** Frequency table for advisors’ own and perception of their students’ career preferences.

	Non-academic	Teaching	Research	Total
	*N*(%)*	*N*(%)	*N*(%)	*N*
Advisors’ general career preference for PhD students		27 (11.1)	12 (4.9)	205 (84)	244
Advisors’ career preference for their specific students				
Student A	80 (38.6)	37 (17.9)	90 (43.5)	207
Student B	58 (36.0)	25 (15.5)	78 (48.4)	161
Student C	35 (35.4)	20 (20.2)	44 (44.4)	99
Advisors’ perception of their students’ career preference				
Student A	76 (36.2)	36 (17.1)	98 (46.7)	210
Student B	64 (42.7)	17 (11.3)	69 (46.0)	150
Student C	27 (28.1)	16 (16.7)	53 (55.2)	96

* Note that % excludes missing cases.

We next turned to an examination of how faculty felt about their students in particular. We expected that when considering *specific* students currently in their labs, STEM faculty would report more balanced career preferences. This was supported by the responses of the faculty (see rows 2–4, Table 5). When asked to consider career preference for their three most senior students, the most commonly chosen option was research (ranging from 43.5 to 48.4%), then non-academic (ranging from 35.4 to 38.6%), followed by teaching (ranging from 15.5 to 20.2%).

These preferences of the faculty closely mirror the proportions that the faculty reported when asked what their perceptions were of their students’ career goals (rows 5–7, Table 5), with the most commonly chosen option being research careers (ranging from 46.0 to 55.2%), then non-academic careers (ranging from 28.1 to 42.7%), followed by teaching careers (ranging from 11.3 to 17.1%). Together, these results suggest that while faculty may ideally prefer to train students who follow paths in academic research similar to their own, they adjust these preferences when considering the skills and interests of particular students.

**Figure 5:**
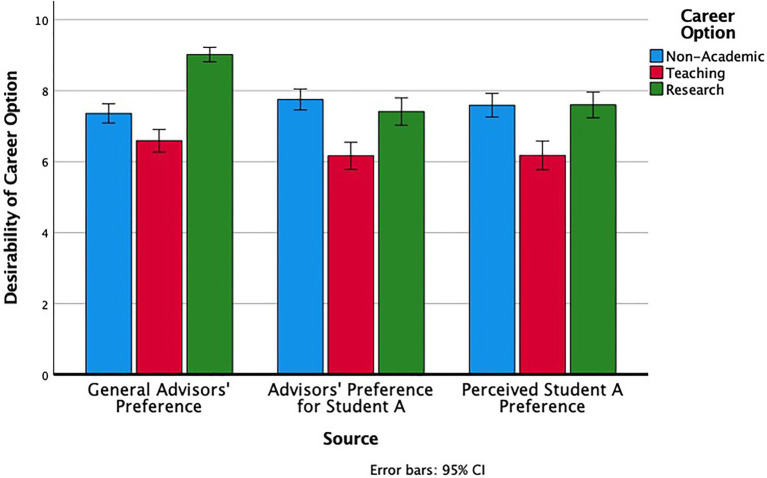
Advisors’ general desirability ratings for each career option (left), desirability ratings for each career option for Student A (their most senior PhD student; middle), and their perception of Student A’s desirability ratings for each career option (right).

The continuous ratings of the desirability of the three positions on the 0 to 10-point scale confirmed this basic pattern. We conducted a 3 (Source: General Advisor’s Preference vs. Advisors’ Preference for Student A vs. Perceived Student A Preference) × 3 (Option: Non-Academic vs. Teaching vs. Research) Repeated Measures ANOVA with both factors within-subjects, and it revealed a significant interaction, *F* (4, 972) = 24.12, *p* < 0.001, ηp2 = 0.09. As Figure 5 shows, for General Advisor’s Preference, research (*M* = 9.00, *SD* = 1.45) was seen as more desirable than non-academic (*M* = 7.31, *SD* = 1.93), pairwise comparison, *p* < 0.001, which in turn was more desirable than teaching positions (*M* = 6.66, *SD* = 2.18), pairwise comparison, *p* < 0.001. By contrast, for Advisor’s Preference for Student A, there was no difference between research (*M* = 7.41, *SD* = 2.78) and non-academic (*M* = 7.58, *SD* = 2.20), pairwise comparison *p* = 0.504, which were both higher than teaching (*M* = 6.42, *SD* = 2.68), both pairwise comparisons *p* < 0.001. Similarly, for Perceived Student A Preference, there was no difference between research (*M* = 7.37, *SD* = 2.84) and non-academic (*M* = 7.41, *SD* = 2.47), pairwise comparison *p* = 0.882, which were both higher than teaching (*M* = 6.27, *SD* = 2.87), both pairwise comparisons *p* < 0.001. Similar interactions and patterns of results occurred for faculty perceptions of Student B, *F* (4, 740) = 19.61, *p* < 0.001, and of Student C, *F* (4, 468) = 12.16, *p* < 0.001, which are presented in Supplementary Material. Although faculty had a clear preference for their students to pursue research careers in general, when considering a specific student, they were more balanced in their career preferences between non-academic and research positions.

#### Predicting Preferences for PhD Students

We next examined whether faculty career preferences for specific students would be driven more by their perception of the *student’s* preference or by their own general preferences. We present a detailed analysis for the faculty of their most senior PhD student (Student A). Table 6 presents two *χ*^2^ analyses. The top cross-tabulation indicates that there is no correspondence between the advisors’ general preferences and the advisors’ preferences for Student A, *χ*^2^ (4, *N* = 172) = 4.57, *p* = 0.33. Examining the diagonal (bolded) indicates that within the category of advisors’ general preferences, there is modest concordance. By contrast, the bottom cross-tabulation indicates strong concordance between the advisors’ perceived Student A preference and the advisors’ preference for Student A, *χ*^2^ (4, *N* = 173) = 142.05, *p* < 0.001. Examining the diagonal (bolded) indicates that within the category of advisors’ perceived preference of student A, there is very strong concordance. In short, while advisors preferred research in general, this does not appear to be what is most predictive of their preference for Student A, but rather, what they perceive as student A’s preference. In Supplementary Material, we present the same *χ*^2^ analysis for Student B and Student C. In each case, the analysis indicates much stronger concordance between the advisors’ perceived student preference and the advisors’ preference for the student.^4^

**Table 6:** Advisor’s own career preference for students in general and advisors’ perception of student A’s (most senior PhD student’s) career preference.

			Advisor’s preference for student A (Rank)			
			Non-academic	Teaching	Research	Total
Advisor’s general career preference	Non-academic	Count	**10**	2	4	16
		%	**62.5**	12.5	25.0	100
	Teaching	Count	3	**1**	4	8
		%	37.5	**12.5**	50.0	100
	Research	Count	53	27	**68**	148
		%	35.8	18.2	**45.9**	100
	Total	Count	66	30	76	172
Advisor’s perception of student A’s career preference	Non-academic	Count	**53**	4	6	63
		%	**84.1**	6.3	9.5	100
	Teaching	Count	3	**22**	7	32
		%	9.4	**68.8**	21.9	100
	Research	Count	12	7	**59**	78	%	15.4	9.0	**75.6**	100	Total
	Count	68	33	72	173

% refers to percentage within general advisor’s preference (for top) and within advisor’s perceived Student A preference (for bottom). Bold numbers indicate concordance between row and column

Next, we examined the continuous preference rating for Student A, and these analyses provide convergent evidence for the categorical results above. We conducted three regression analyses where the outcome variable was advisors’ preferences for Student A to pursue each career option (non-academic top; teaching middle; research bottom), and the two predictors were their general preference and their perception of the students’ preferences, all on the continuous scale (see Table 7). In each regression, the strength of the perceived student preference was much stronger (non-academic standardized *ß* = 0.67 vs. 0.31; teaching standardized *ß* = 0.70 vs. 0.29; research standardized *ß* = 0.70 vs. 0.09). In sum, while general preferences of advisors play a role in what they prefer for their most senior student, their preferences were much more strongly driven by what they perceive the student as preferring for their career. Similar findings were obtained for Student B and Student C (see Supplementary Material).

**Table 7:** Determinants of advisors’ career preferences for student A.

Outcome variables: advisors’ desirability ratings for career options for student A	*b*	*SE*	𝛃	*t*	*p*
Non-academic	Constant	0.58	0.33		1.79	0.08
	Advisor’s desirability ratings for non-academic positions for their students in general	0.36	0.04	0.31	8.23	<0.001
	Perceived student A’s desirability ratings for non-academic positions	0.59	0.03	0.67	17.45	<0.001
Teaching	Constant	−0.06	0.29		−0.19	0.85
	Advisor’s desirability ratings for teaching positions for their students in general	0.36	0.04	0.29	8.09	<0.001
	Perceived student A’s desirability ratings for teaching positions	0.66	0.03	0.70	19.66	<0.001
Research	Constant	0.74	0.78		0.95	0.34
	Advisor’s desirability ratings for research positions for their students in general	0.18	0.09	0.09	2.07	0.04
	Perceived student A’s desirability ratings for research positions	0.69	0.05	0.70	15.39	<0.001

#### Comfort in Discussing Different Career Options With PhD Students

We conducted a repeated measures ANOVA to examine how STEM faculty advisors felt about advising their students in regard to the different career options. In particular, we examined whether they had varying levels of comfort discussing the three different options. The repeated measures ANOVA revealed a significant difference, *F* (2, 548) = 64.98, *p* < 0.001, ηp2 = 0.19, as, consistent with the perceptions of students, faculty advisors felt more comfortable discussing research careers (*M* = 9.36, *SD* = 1.03) than non-academic positions (*M* = 8.17, *SD* = 1.97), *p* < 0.001, or teaching focused positions (*M* = 8.16, *SD* = 2.12), *p* < 0.001, with no difference between teaching and non-academic, *p* = 0.96.

#### Faculty Advisors’ Perceptions of Their Students’ Belonging

Faculty advisors rated their perception of belonging levels within their academic departments for the same three most senior students, which provides the opportunity to see how faculty advisors assess their students as a function of how they perceived their career trajectories. We transposed the data so that each student (*N* = 614) is an individual case, however, multiple students were reported by individual advisors (122 faculty members rated three students, 69 faculty members rated two students, and 110 faculty members rated one student). Faculty advisors reported what they thought each of those students would rank highest in terms of career preference for themselves; 167 students were perceived as ranking non-academic positions highest, 68 students were perceived as ranking teaching positions highest, and 220 students were perceived as ranking research positions highest, with 159 missing (possibly because faculty did not know and left that blank). We also asked faculty advisors to report each student’s race/ethnicity, from which we coded the students as non-URM (Asian/Asian Americans, White/European Americans, *N* = 418) and URM (Black/African American; Hispanic/Latino American; Native American; Pacific Islander; Multi-Racial/URM, *N* = 100; Other, *N* = 41; Missing/Unspecified = 55) and perceived FG status (first in family to attend college, *N* = 101; not first in generation to attend college, *N* = 282; Not sure, *N* = 177; Missing/Unspecified, *N* = 54).

**Figure 6:**
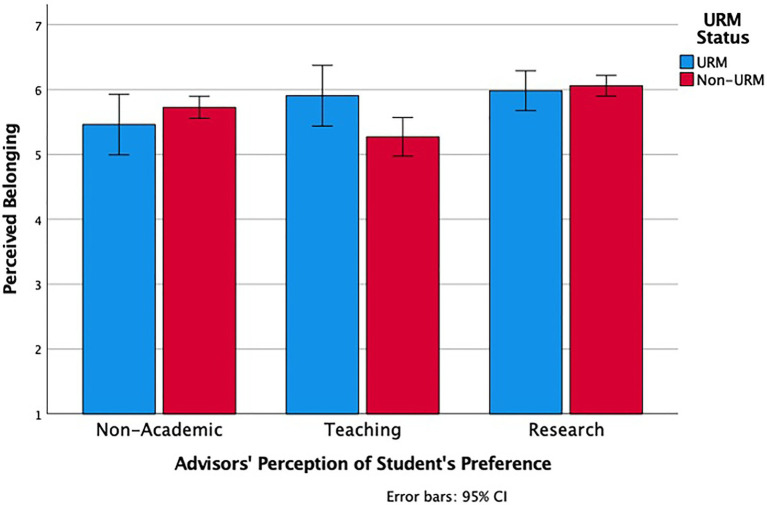
Advisors’ perception of their students’ sense of belonging, as a function of URM status and their perception of students’ career preference.

We conducted an ANOVA to examine faculty advisors’ perceptions of their students belonging in the department, as a function of their perceived top career choice (Non-Academic, Teaching, Research) and their demographic status as URM (vs. non-URM). Overall, there was a main effect of career choice, *F* (2, 404) = 5.53, *p* = 0.004, ηp2 = 0.027, as students perceived to prefer research were seen as belonging more in the academic department (*M* = 6.04, *SD* = 0.94) than those who prefer non-academic (*M* = 5.70, *SD* = 0.99), pairwise comparison *p* = 0.002, or those who prefer teaching (*M* = 5.46, *SD* = 1.29), pairwise comparison *p* < 0.001. There was no main effect of URM status, *F* (1, 404) = 0.49, *p* = 0.49, as faculty perceived URM students to feel just as much belonging (*M* = 5.84, *SD* = 1.06) as non-URM students (*M* = 5.82, *SD* = 1.03). This is noteworthy, considering that URM students felt less belonging than non-URM students, as reported in Study 1. There was also an interaction, *F* (2, 404) = 3.15, *p* = 0.044, depicted below in Figure 6. The interaction appears to be driven by perceptions of the non-URM students’ belonging, as it was seen to be much higher for those interested in research (*M* = 6.06, *SD* = 0.88) than either teaching, (*M* = 5.27, *SD* = 1.39), *p* < 0.001, or non-academic careers (*M* = 5.73, *SD* = 0.98), *p* = 0.006. By contrast, there was no significant difference for URM students in their perceived belonging between teaching (*M* = 5.91, *SD* = 0.91) and research (*M* = 5.98, *SD* = 1.11), *p* = 0.79, with only non-academic being somewhat less than research (*M* = 5.46, *SD* = 1.04), *p* = 0.068.

**Figure 7:**
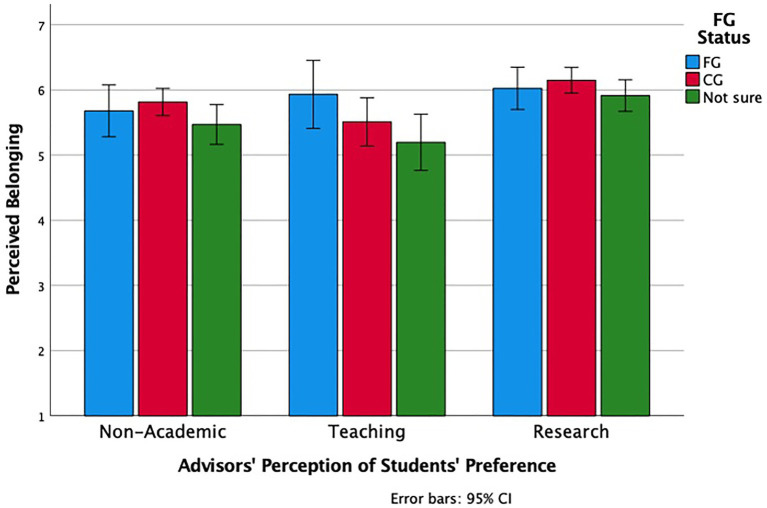
Advisors’ perceived belonging of their students, as a function of first-generation (FG) status and their perception of students’ career preference.

We conducted a similar ANOVA to examine faculty advisors’ perceptions of their students, belonging in the department as a function of their perceived top career choice (Non-Academic, Teaching, Research) and the perceived FG status of the PhD student. As reported above, there was a main effect of career choice. There was also a main effect of generation status, *F* (2, 436) = 3.54, *p* = 0.03, ηp2 = 0.02. Interestingly, when faculty advisors reported that they were not sure of the generation status of the PhD students they were advising, they perceived that student as belonging less (*M* = 5.65, *SD* = 1.17) than either students who they perceived as first in the family to go to college (*M* = 5.90, *SD* = 0.99), *p* = 0.09 or not first in the family to go to college (*M* = 5.93, *SD* = 0.99), *p* = 0.01. There was no interaction between the variables, *F* (4, 436) = 0.78, *p* = 0.54 (see Figure 7).

### Discussion

In Study 2, we explored faculty advisors’ career preferences for PhD students, both in general and for specific students they are advising. Through both categorical and continuous measures, we found that faculty advisors, in general, preferred their students to pursue research-focused academic positions. In this way, their preferences were consistent with what the PhD students perceived in Study 1. However, when thinking of a specific student, faculty advisors reported a more balanced career preference, and the patterns were similar to their perception of that student’s career goals. This appears to be discrepant with the PhD students’ perceptions in Study 1, the majority of whom thought that their advisors preferred them, specifically, to pursue research careers, even though they were more balanced in their career goals. Of course, because these are two separate samples, we are cautious in our interpretation about claims of “accuracy” but it does appear that what PhD students perceived in Study 1 to be true of their advisors’ goals for them corresponds better to STEM advisors’ abstract goals, than the goals that STEM advisors’ report having for their specific students.

We further examined whether the patterns in career preferences were more strongly related to advisors’ own general preferences or to their perception of the preferences of students they advise. We found that faculty advisors’ career preferences for specific students they advise were primarily driven by their perception of the student’s career preference instead of their own general preference. In other words, according to their own assessments, faculty advisors do not generally impose their self-preference when thinking about the career development of students they advise. Instead, they seemed to orient their mentorship to what they perceived that the PhD students preferred. We then examined how comfortable faculty advisors are in advising students with regard to the different career options. The patterns mirror the students’ perception from Study 1. Faculty advisors were significantly more comfortable discussing research positions than non-academic or teaching positions with students they advise.

Faculty advisors who perceived the top career preference of students they advise to be research rated those students as experiencing more belonging in the department, compared to those who preferred non-academic or teaching positions. In considering students’ demographic characteristics, faculty advisors did not differ in their overall perception of the sense of belonging experienced by URM and non-URM students. This finding is important to consider as it contrasts with the students’ feelings of belonging in Study 1, where non-URM students felt more belonging in the departments than URM students. However, faculty viewed non-URM students interested in research as experiencing much more belonging than those interested in teaching and non-academic positions. There was no significant difference for URM students with different career preferences. Finally, faculty advisors who were uncertain about their students’ generation status perceived them as feeling less belonging compared to FGs and CGs, regardless of their career preferences. This unexpected finding may be due to a third variable, such as closeness between advisor and student; advisors who are less close to students may not know them (and details such as generation status) as well and perceive them as belonging less.

## General Discussion

Integrating the results of Study 1 and Study 2 suggests that STEM PhD students are accurate in their perception that faculty, in general, would prefer students in their labs to pursue research-focused academic positions. They are also accurate in their perception that faculty advisors are more comfortable discussing research careers than other options. Faculty, by contrast, perceive themselves as being aligned with graduate students’ preferences in their post-PhD pursuits, regardless of which of the three directions they believe the student desires to pursue. When faculty think about specific students, they adjust their goals to align with what they perceive the students’ goals to be.

Such adjustments, of course, may not be explicitly communicated to PhD students by their advisors. When students realize that they are interested in pursuing non-academic careers, they may see themselves as misaligned with their advisors’ interest, and they may persist in thinking that their advisors want them to pursue research. These students more interested in non-research careers are discrepant from what they see as normative in their departments, and this discrepancy is associated with students feeling a lack of belonging and social support, factors which are associated with academic performance (Cohen and Garcia, 2008).

### Interpersonal and Institutional Implications

The present findings suggest several implications on the interpersonal (i.e., faculty advisor-student) and institutional (i.e., departmental and university) levels. First, more discussions about students’ career development should be encouraged, and faculty should be more explicit and open about their support for students’ various career preferences. Although students may have initially expressed interest in pursuing research careers, some students preferences change over time, and they may not be comfortable revealing such changes to their advisors (Sauermann and Roach, 2012). Approximately 20% of the PhD students in Study 1 indicated that teaching was their career preference, which would lead them to join the professoriate, and yet they felt a lower sense of belonging than those who preferred research-oriented careers, a finding with implications for diversifying the professoriate. Having more regular conversations about career development and knowing about their advisors’ support may increase students’ comfort in having discussions about pursuing teaching and non-academic career paths and, therefore, their ability to pursue and secure resources to help them reach those goals. The present findings suggest that creating individual development plans (IDP; Austin and Alberts, 2012) between faculty advisors and doctoral students in the sciences would be helpful, particularly if they are regularly revisited as students progress through their academic programs.

Second, faculty may not be fully aware of how demographic characteristics of PhD students affect their sense of belonging and perceived social support. Faculty advisors in Study 2 did not perceive any difference in belonging for URM students compared to non-URM students, whereas PhD students in Study 1 reported such a discrepancy. This discrepancy across studies could be due to many factors, including inaccurate perceptions of faculty, or the faculty participants in our studies were assessing a different sample of participants as those who participated in Study 1. Research that includes dyadic assessments (where advisors and students participate as pairs) would help clarify this discrepancy. When examining faculty members’ perception of students’ belonging as a function of their generation status, faculty who were unsure of the college generation status of students they advised perceive them as experiencing less belonging. For faculty who know about the college generation status of students they advise, their perceptions of the students’ belonging mirrored the student experiences, such that FGs felt less belonging than CGs. Although we did not find any significant difference in FGs and CGs’ perceived social support from their advisors (in Study 1), understanding students’ demographic backgrounds can inform faculty members about ways they can provide social support to bolster FGs’ and URMs’ sense of belonging. Mentorship training and institutional practices that incorporate greater discussion of backgrounds may be particularly useful, such as the University of California’s program that highlights FG status of students and faculty alike (University of California, 2021).

At the institutional level, we examined norms within the academic department, where students receive advising and interact with multiple faculty members. Overall, PhD students in Study 1 perceived a strong normative preference from their advisors and other faculty members for pursuing research careers. While students are accurate in reflecting the general preference of faculty members, they may not recognize that faculty can be responsive (and see themselves as wanting to be responsive) toward student preferences (as indicated in Study 2), thus missing out on potential opportunities to discuss alternative career paths with their advisors and mentors, including teaching positions which would enable them to continue on the path to the professoriate. The norms that faculty communicate informally to students can influence their psychological experience and choices.

### The Power of Perceptions

A recent study shows the power of the perceived beliefs of STEM faculty members (LaCosse et al., 2020). The researchers had students evaluate STEM courses that were taught by professors who, via random assignment, either expressed fixed or growth mindset beliefs about intelligence. Students anticipated more negative experiences in the classes purportedly taught by faculty who believed that intelligence is fixed than those taught by faculty who believed intelligence is malleable, anticipating that they would perform worse in such classes and exhibiting less interest in taking them (LaCosse et al., 2020). This pattern occurred for all students but was particularly strong for the female STEM students.

In the present research, it appears that PhD STEM students may perceive that faculty in their departments, including their advisors, have a relatively fixed view on what career is most desirable (research), whereas the faculty view themselves as possessing a more malleable view that is adaptive to their students’ needs. We suspect that students’ perceptions of faculty advisors’ preferences as relatively stable may lead them to feel less efficacious about discussing other options. We also suspect that to the extent that faculty advisors see themselves as malleable in their career preferences for students, this malleability may not be explicitly communicated to students. Together, it may be advisable for both parties to have structured career discussions and to normalize and explicitly signal support for diverse careers paths.

Prior studies revealed that PhD students in highly structured STEM doctoral programs (e.g., that promoted an early and systematic involvement in research) with explicit publication expectations had fewer publication gaps between URMs (vs. non-URMs) and women (vs. men; Mendoza-Denton et al., 2017). What the authors describe as a “culture of structure” and a clearly outlined path led to greater success for a wider range of students (Fisher et al., 2019). Approaches that foster a culture of structure to help emphasize the support available to pursue diverse career paths can potentially be incorporated into doctoral students’ career development. A culture of structure may be beneficial to faculty members as well, with clearly defined benchmarks for advising. Departments can signal their support for diverse career options as well and take actions to promote them by integrating career exploration into doctoral program milestones, encouraging summer internships, and holding workshops for teaching and non-academic career preparation. This may require more training of faculty to communicate that career development is part of mentoring and provide them with resources to mentor their students on non-research careers.

### Limitations

Several limitations in this research warrant mention. First, although we investigated career preferences from both students’ and faculty advisors’ perspectives, we did not have a matched sample. We would like to emphasize that we cannot assess the accuracy of their different perceptions in the current research (as we could in a design that examined PhD student-advisor dyads). We cannot verify the accuracy of the students’ views in Study 1 (students may be incorrect in what their advisors’ preferences are for them), nor can we verify the accuracy of advisors’ views in Study 2 (advisors may be incorrect in their assumptions of what career path the students they advise wish to take). Future research examining advisor-mentee dyads would be useful to understand the communicative context more clearly. Second, we did not have sufficient sample size to explore the variability between different URM groups or examine the intersectional relationship between race/ethnicity and gender. Moreover, we did not have a sufficient sample size to examine how variability amongst the STEM disciplines influences the perceptions of students and faculty alike. Given the still-limited ethnic and racial diversity in many graduate programs, a larger study undertaken across many more universities in order to develop a complete picture of these important layers could reveal a more nuanced and detailed picture. STEM areas have a varying degree of focus on applied (vs. basic) research, which can manifest in different attitudes and connections with institutions and organizations outside academia. For example, researchers in computer science have more connections and collaborations with technology companies, whereas those in some basic sciences may lack those connections and knowledge of research conducted beyond academia and the potential for diverse professional paths within those fields. A closer examination of the attitudes toward non-academic careers will help better identify fields that have stronger norms and preferences toward academic and non-academic careers.

### Closing Thoughts

From the perspective of the PhD student in STEM fields, career development is an integral part of their doctoral studies. The present studies highlight an asymmetry between perceived and actual norms in career preferences from the students’ and faculty advisors’ perspectives and point out that faculty may not be as unsupportive toward teaching and non-academic careers as students may perceive them to be. Having more explicit and frequent conversations at both the interpersonal and institutional levels can not only address such asymmetries, but more importantly, may also create a more welcoming and supportive academic environment that is attuned to the contemporary constraints and opportunities in academia and industry.

## Data Availability Statement

The datasets presented in this study can be found in online repositories. The names of the repository/repositories and accession number(s) can be found at: https://osf.io/4uyxh/.

## Ethics Statement

The studies involving human participants were reviewed and approved by UCSB ORAHS 61-21-0256. The patients/participants provided their written informed consent to participate in this study.

## Author Contributions

DS developed and executed the research described in the paper and analyzed the data. LO executed the research plan and collected the data. SL analyzed the data. DS, LO, and SL wrote the manuscript. CK and MH commented on the manuscript and helped to organize the data collection. All authors contributed to the article and approved the submitted version.

## Funding

Collaborative research: the Alliances for Graduate Education in the Professoriate (AGEP) California Hispanic Serving Institutions (HSI) Alliance to Increase Underrepresented Minority Faculty in STEM. National Science Foundation Award #1820886.

## Conflict of Interest

The authors declare that the research was conducted in the absence of any commercial or financial relationships that could be construed as a potential conflict of interest.

## Publisher’s Note

All claims expressed in this article are solely those of the authors and do not necessarily represent those of their affiliated organizations, or those of the publisher, the editors and the reviewers. Any product that may be evaluated in this article, or claim that may be made by its manufacturer, is not guaranteed or endorsed by the publisher.

## Footnotes

^1^We use the term URM students to refer to students whose racial/ethnic group are underrepresented in STEM disciplines. NSF has identified that Blacks, Hispanics, and Native Americans/Native Alaskans are under-represented in science and engineering professions National Science Foundation (2017) and we use the term in this specific sense.

^2^Sample size was determined based on analysis of graduate student demographics to enable adequate representation students from engineering, natural science, physical sciences, and social sciences as well as URM. We targeted *N* = 250 and had 195 complete all measures. Multiple recruitment notices were sent out by graduate division at each university.

^3^Sample size goal of *N* = 300 was determined by the goal of seeking disciplinary balance across engineering, natural science, physical sciences, social sciences across the two campuses with a minimum of *N* = 50 per discipline. Multiple recruitment emails were sent to faculty to try to achieve this sample. The percentage of faculty who participated was 301/692 overall (43.4%), 177/525 for UCSB (33.7%), and 97/167 for UCM (58.1%). More detailed descriptions on samples and fields of study are described in supplemental materials on the study link on Open Science Framework.

^4^The pattern is very similar for Student C, whereas, for Student B, there is also significant concordance between general preference and preference for Student B, although the concordance is weaker than with the perceived preference of Student B and faculty preference for Student B.

## Supplementary Material

The Supplementary Material for this article can be found online at: https://www.frontiersin.org/articles/10.3389/fpsyg.2021. 711615/full#supplementary-material
