# Supplementary material for: The Changing Landscape of Doctoral Education in Science, Technology, Engineering, and Mathematics: PhD Students, Faculty Advisors, and Preferences for Varied Career Options
Source: Front Psychol. 2021 Dec 17;12:711615. doi: 10.3389/fpsyg.2021.711615 (PMC8771201; doi:10.3389/fpsyg.2021.711615)
Supplement: Supplementary file 1 [file Data_Sheet_1.docx]

**Supplementary Materials**

**Study 1 Supplemental Measures**

*Open-Ended Questions.* Participants were asked to elaborate on why they were comfortable or uncomfortable in discussing the three career categories with their advisors. Specifically, they were asked to respond to the prompt “*Please think about having discussions with your advisor about non-academic positions (e.g., industry, government, non-profit organization). To what extent would you feel comfortable in these discussions, and why?*” The same questions were asked for teaching and research focused positions.

*Hypothetical Scenarios*. Participants were asked to rank their priorities for professional development in several hypothetical scenarios. The scenarios were common to most doctoral students’ experience (e.g., summer professional development plans, attending conference panels, and attending on-campus professional development workshops). Participants were asked to prioritize the types of opportunities and skillsets they hope to develop most. For example, in the summer plans scenario, participants were asked which of the three options – getting an internship in a non-academic organization, teaching undergraduate summer courses, and working on academic research – they would prioritize during the summer. The same set of questions and scenarios were asked about their perceived advisors’ perspective.

*Mentorship Ability & Motivation*. Participants were asked how capable they felt their advisor would be at mentoring them for each of the three career categories, as well as how motivated they were to do so. Each career category was assessed separately, with responses from 0 (not at all capable / not at all motivated) to 10 (extremely capable / extremely motivated).

**Study 1 Supplemental Results**

*PhD students’ Perceived Desirability for Different Career Options as a Function of Underrepresented Minority (URM) and First-Generation College Student (FG) Status*


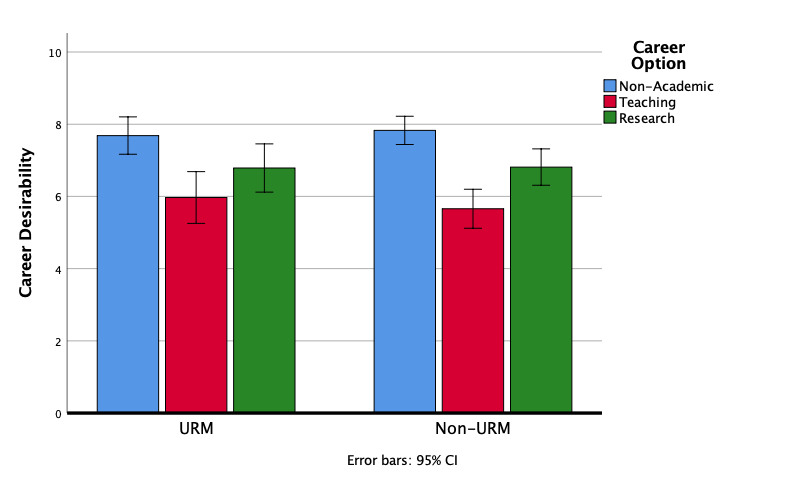


**Figure 1.** URM status and career desirability. A 3 (Career Option: Non-Academic, Teaching, Research) x 2 (URM Status: URM, non-URM) with career desirability as a dependent variable mixed-model ANOVA revealed a main effect of career option on career desirability, *F*(2, 382) = 19.4, *p* <.001, *η*_p_^2^ = .09. There was no main effect of URM status *F*(1, 191) = .06, *p* = .80, *η*_p_^2^ <.001, and no interaction between the two variables, *F*(2, 382) = .29, *p* = .75, *η*_p_^2^ = .01.


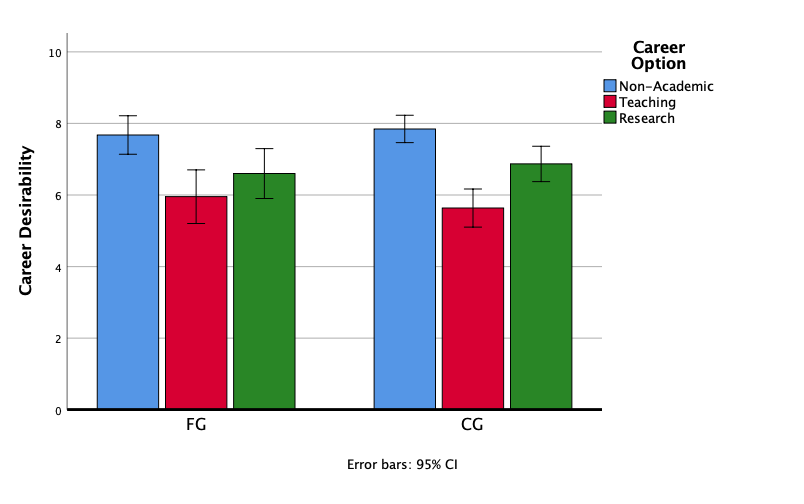


**Figure 2.** College generation status and career desirability. A 3 (Career Option: Non-Academic, Teaching, Research) x 2 (FG Status: FG, CG) with career desirability as the dependent variable mixed-model ANOVA revealed a main effect of career option on career desirability, *F*(2, 384) = 19.17, *p* <.001, *η*_p_^2^ = .09. There was no significant main effect of FG status *F*(1, 192) = .04, *p* = .84, *η*_p_^2^ <.001, and no interaction between the two variables, *F*(2, 384) = .49, *p* = .62, *η*_p_^2^ =.003.


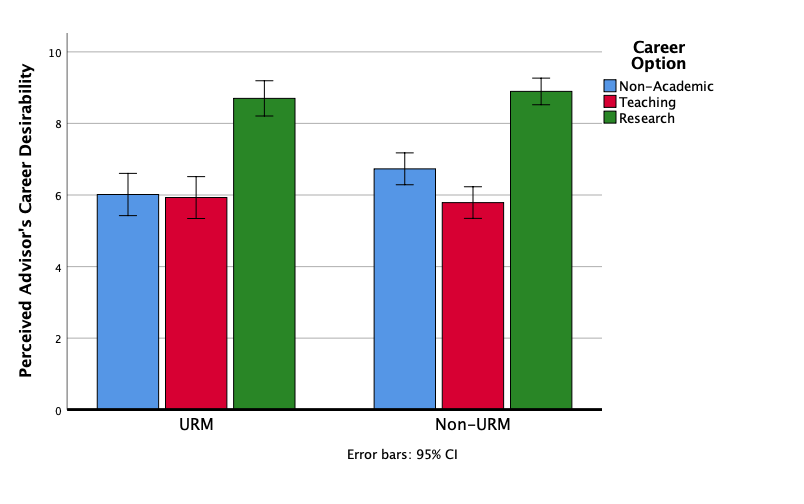


**Figure 3.** URM Status and Perceived Advisor’s Career Desirability. A 3 (Career Option: Non-Academic, Teaching, Research) x 2 (URM Status: URM, Non-URM) mixed-model ANOVA predicting perceived advisor’s career desirability for them to pursue each career revealed a significant main effect of career option on career desirability, *F*(2, 382) = 75.08, *p* <.001, *η*_p_^2^ = .28. There was no significant main effect of URM status *F*(1, 191) = 1.74, *p* = .19, *η*_p_^2^ =.01, and no interaction between the two factors, *F*(2, 382) = 1.42, *p* = .24, *η*_p_^2^ =.01.


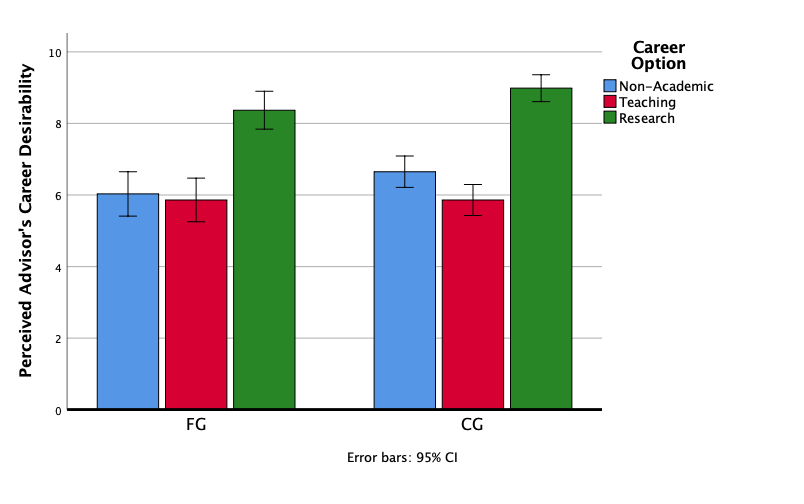


**Figure 4.** Generation Status and Perceived Advisor’s Career Desirability. A 3 (Career Option: Non-Academic, Teaching, Research) x 2 (FG Status: FG, CG) mixed-model ANOVA predicting perceived advisor’s desirability for them to pursue each career revealed a significant main effect of career option on career desirability, *F*(2, 384) = 64.60, *p* <.001, *η*_p_^2^ = .25. There was also significant main effect of FG status, *F*(1, 192) = 4.24, *p* = .04, *η*_p_^2^ =.02, but no significant interaction between the two factors, *F*(2, 384) = .91, *p* = .40, *η*_p_^2^ =.01. FGs (*M* = 8.37, *SD* = 2.60) rated a marginally lower perceived advisors’ career desirability for them to pursue research careers than did CGs (*M* = 8.98, *SD* = 1.92, *p* = .06).

*Relationship between Career Preferences and Belonging as a Function of Demographic Characteristics*

*
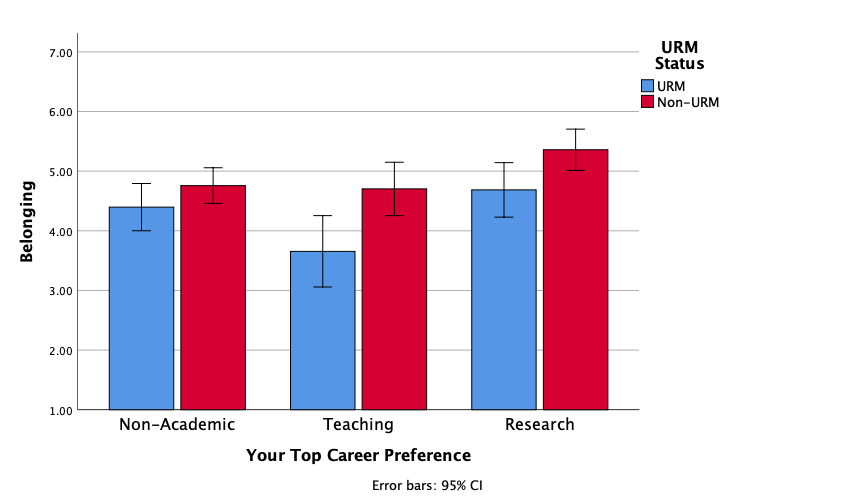
*

**Figure 5.** Function of URM Status on the Relationship between Career Preference and Belonging. A 3 (Career Preference: Non-Academic, Teaching, Research) x 2 (URM Status: URM, Non-URM) between-subjects ANOVA with belonging as the dependent variable revealed a main effect of career preference, *F* (2, 187) = 6.54, *p* = .002, *η*_p_^2^= .07, and a main effect of URM Status, *F*(1, 187) = 14.85, *p* <.001, *η*_p_^2^ = .07. There was no interaction between the two factors, *F* (2, 187) = 1.18, *p* = .31, *η*_p_^2^ = .01.


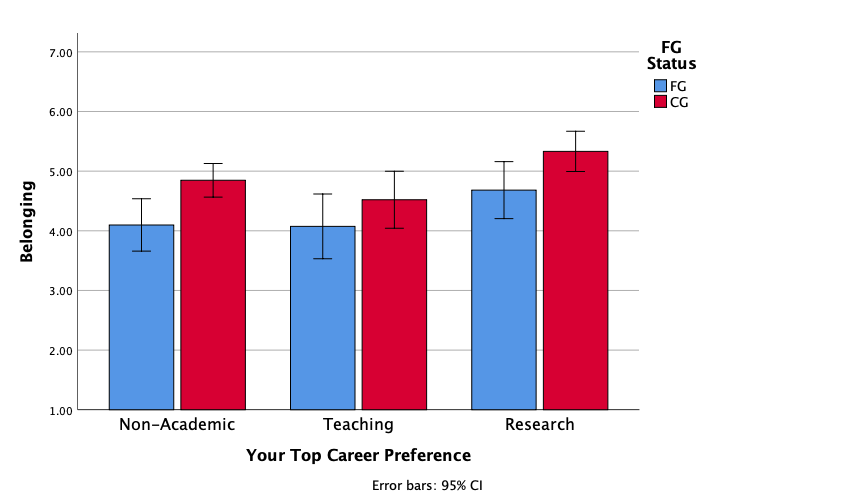


**Figure 6.** Function of FG Status on the Relationship between Career Preference and Belonging. A 3 (Career Preference: Non-Academic, Teaching, Research) x 2 (FG Status: FG, CG) between-subjects ANOVA with belonging as the dependent variable revealed a main effect of career preference, *F* (2, 188) = 5.57, *p* = .004, *η*_p_^2^ = .06, and a main effect of FG Status, *F*(1, 188) = 11.65, *p* =.001, *η*_p_^2^ = .06. There was no interaction between the two variables, *F* (2, 188) = .23, *p* = .80, *η*_p_^2^ = .01.

*Relationship between Career Preferences and Perceived Social Support as a Function of Demographic Characteristics*

*
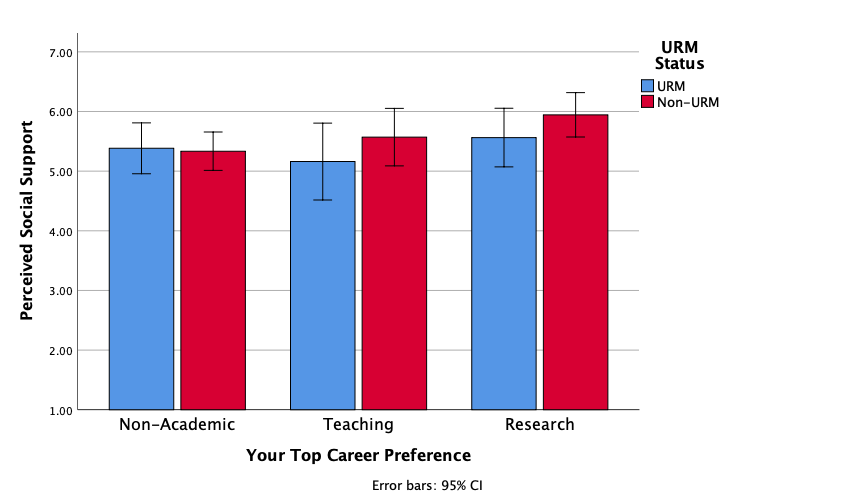
*

**Figure 7.** Function of URM Status on the Relationship between Career Preference and Perceived Social Support. A 3 (Career Preference: Non-Academic, Teaching, Research) x 2 (FG Status: FG, CG) between-subjects ANOVA with perceived social support as the dependent variable revealed no main effect of career preference, *F* (1, 187) = 2.07, *p* = .13, *η*_p_^2^ = .02, and no main effect of URM Status, *F*(1, 187) = 1.63, *p* =.20, *η*_p_^2^= .01. There was no interaction between the two variables, *F* (1, 187) = .72, *p* = .49, *η*_p_^2^ = .01.


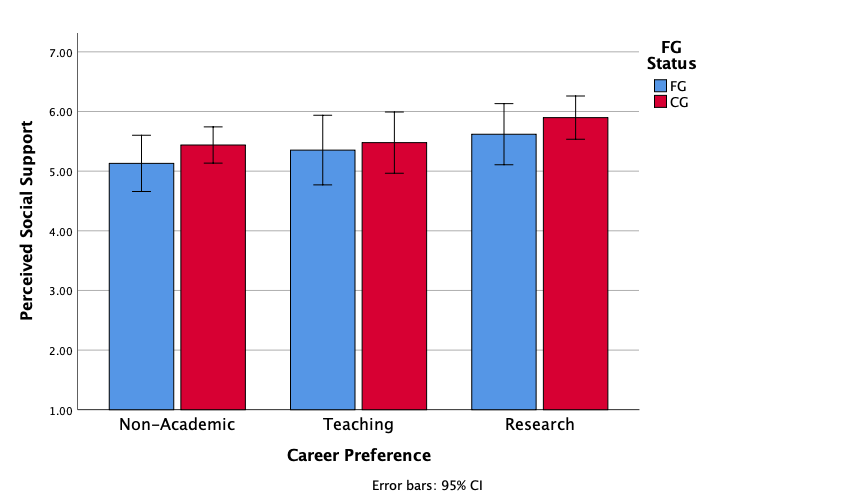


**Figure 8.** Function of FG Status on the Relationship between Career Preference and Perceived Social Support. A 3 (Career Preference: Non-Academic, Teaching, Research) x 2 (FG Status: FG, CG) between-subjects ANOVA with perceived social support as the dependent variable revealed no main effect of career preference, *F* (1, 188) = 2.53, *p* = .08, *η*_p_^2^ = .03, and no main effect of FG Status, *F* (1, 188) = 1.50, *p* =.22, *η*_p_^2^ = .008. There was no interaction between the two variables, *F* (2, 188) = .08, *p* = .93, *η*_p_^2^ = .001.

*Comfort in Discussing Each Career Option as a Function of Demographic Characteristics*

*
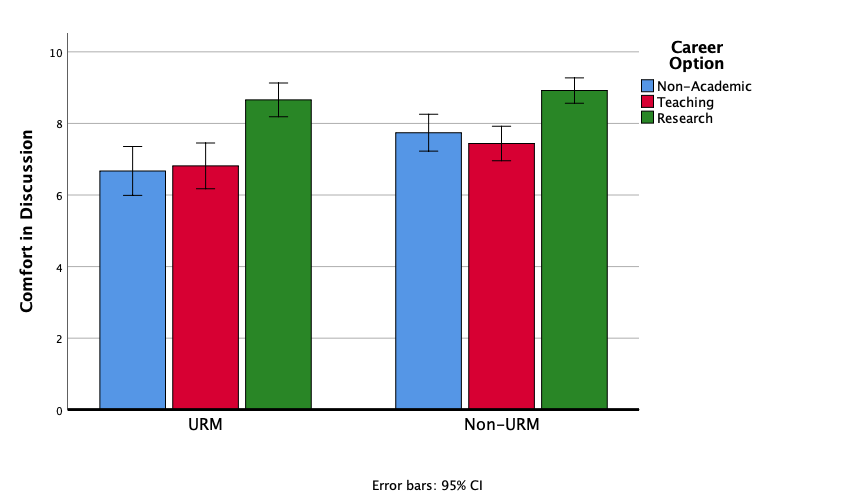
*

**Figure 9.** Function of URM Status on comfort in discussing each career option. A 3 (Career Option: Non-Academic, Teaching, Research) x 2 (URM Status: URM, non-URM) Repeated Measure ANOVA with comfort in discussion as the dependent variable revealed a main effect of career option, *F* (2, 382) = 46.11, *p* <.001, *η*_p_^2^ = .19, and a main effect of URM Status, *F* (1, 191) = 4.39, *p* =.04, *η*_p_^2^ = .02. There was no interaction between the two factors, *F* (2, 382) = 2.14, *p* = .12, *η*_p_^2^ = .01.


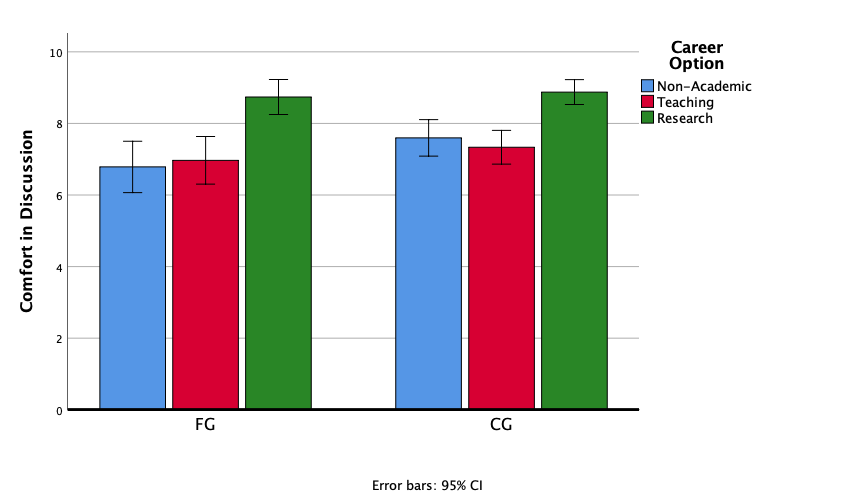


**Figure 10.** Function of FG Status on comfort in discussing each career option. A 3 (Career Option: Non-Academic, Teaching, Research) x 2 (FG Status: FG, CG) Repeated Measure ANOVA with comfort in discussion as the dependent variable revealed a main effect of career option, *F* (2, 384) = 46.69, *p* <.001, *η*_p_^2^ = .19, but no main effect of FG Status, *F* (1, 192) = 1.90, *p* =.17, *η*_p_^2^ = .01. There was no interaction between the two factors, *F* (2, 384) = 1.48, *p* = .23, *η*_p_^2^ = .01.

**Study 2**

**Exclusion Criteria**

A total of 312 participants’ responses were recorded. We excluded participants who did not consent (*N* = 4) and participants who only responded to the first question (*N* = 7), leaving a final sample for analysis of *N* = 301.

**Study 2 Supplemental Measures**

*Capability and Motivation*. All faculty respondents were asked how capable they felt mentoring students who wanted to pursue each of the three career pathways on a scale from 0 (not at all capable) to 10 (extremely capable). They were then also asked how motivated they were to mentor students who wanted to pursue each of the three career pathways, on a scale from 0 (not at all motivated) to 10 (extremely motivated).

*Affective Response.* In the next section, faculty members were asked to recall in detail a time when a student they hoped would pursue a career in academic research told them they actually preferred a career in either an academic teaching role or a non-academic role. If they had never been in such a conversation, they were able to skip this section entirely. For those who had been, they were asked to describe the degree to which they felt a range of emotions measured from 1 (not at all) to 5 (completely).  The emotions they were asked to assess were unhappy, proud, sad, disappointed, pleased, and supportive. These emotions were assessed separately for situations in which a students declared interest in teaching and those in which a student declared interest in a non-academic career.

*Comfort with Advising*. Next, faculty members were asked to assess how comfortable they felt with the part of their job that includes advising students on career paths, responding separately for each of the three options that have been used throughout. Faculty comfort was measured from 0 (very uncomfortable) to 10 (very comfortable).

*Mentorship Goals*. The next section assessed faculty members’ values and priorities in mentoring relationships. These questions were intended to be treated as single items, not a coherent scale, that may inform the primary findings about career preference and belonging. Faculty were asked to consider the degree to which each item was an ‘important goal and responsibility’ in their role as advisor to graduate students. Sample items include, “Train the next generation of researchers,” “Help bright young people reach diverse professional goals,” “Provide emotional support,” and “Provide direct hands-on training in research methods.” The full 15-item list can be found in supplemental materials.

*Professional Information*. The next section contained a variety of questions related to that faculty member’s professional experience both inside and outside of academia. These items included the total number of students they have advised and the total number of years they have worked in academia, as well as information about any work experience they might have *outside* of academia. Faculty members were also asked to provide – to the best of their ability – information about how many of their previous students now work in each of the three fields that have been under consideration throughout.

**Study 2 Supplemental Results**

**
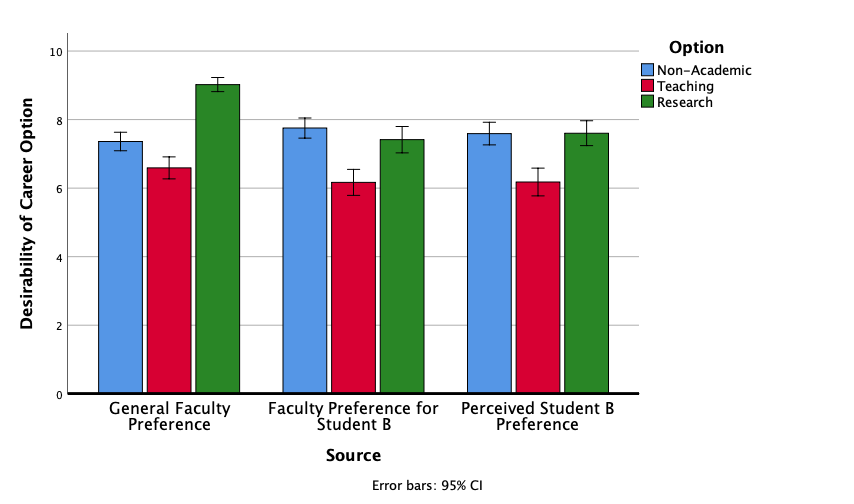
**

**Figure 11.** General faculty preferences for non-academic, teaching, and research careers for their students, faculty preferences for Student B (their second most senior PhD student), along with what faculty perceive as Student B’s preferences among the three options. We conducted a 3 (Source: General Faculty Preference vs. Faculty Preference for Student B vs. Perceived Student B Preference) X 3 (Option: Non-Academic vs. Teaching vs. Research) Repeated Measures ANOVA with both factors within-subjects. We found a significant interaction, *F*(4, 740) = 19.61, *p* <.001, η_p_^2^ = .10. For general faculty preference, research (*M* = 9.02, *SD* = 1.42) was seen as more desirable than non-academic (*M* = 7.36, *SD* = 1.86), pairwise comparison *p* <.001. By contrast, for faculty preference for Student B, there was no difference between research (*M* = 7.41, *SD* = 2.67) and non-academic (*M* = 7.75, *SD* = 2.03), pairwise comparison, *p* = .22, which were both higher than teaching (*M* = 6.17, *SD* = 2.62), both pairwise comparisons, *p* <.001. For perceived student B preference, there was no difference between research (*M* = 7.60, *SD* = 2.51) and non-academic (*M* = 7.59, *SD* = 2.29), pairwise comparison, *p* = .97, which were both higher than teaching (*M* = 6.18, *SD* = 2.80), both pairwise comparisons, *p* <.001.


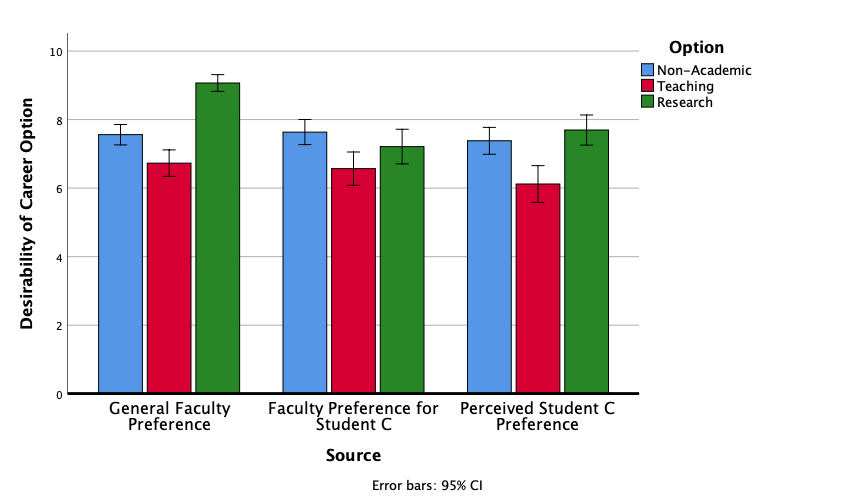


**Figure 12.** General faculty preferences for non-academic, teaching, and research careers for their students, faculty preferences for Student C (their third most senior PhD student), along with what faculty perceive as Student C’s preferences among the three options. We conducted a 3 (Source: General Faculty Preference vs. Faculty Preference for Student C vs. Perceived Student C Preference) X 3 (Option: Non-Academic vs. Teaching vs. Research) Repeated Measures ANOVA with both factors within-subjects. We found a significant interaction, *F*(4, 468) = 12.16, *p* <.001, η_p_^2^ = .10. For general faculty preference, research (*M* = 9.07, *SD* = 1.34) was seen as more desirable than non-academic (*M* = 7.56, *SD* = 1.63), pairwise comparison *p* <.001. By contrast, for faculty preference for Student B, there was no difference between research (*M* = 7.21, *SD* = 2.77) and non-academic (*M* = 7.64, *SD* = 2.00), pairwise comparison, *p* = .22, which were both higher than teaching (*M* = 6.57, *SD* = 2.66), both pairwise comparisons, *p* < .05. For perceived student B preference, there was no difference between research (*M* = 7.69, *SD* = 2.41) and non-academic (*M* = 7.38, *SD* = 2.15), pairwise comparison, *p* = .34, which were both higher than teaching (*M* = 6.12, *SD* = 2.95), both pairwise comparisons, *p* <.001.

| **Table 1** |  |  |  |  |  |  |
| --- | --- | --- | --- | --- | --- | --- |
| *Rank of Self and Perceived Student B (Second Most Senior PhD Student) Career Preference* | | | | | | |
|  |  |  | Advisor's Preference for Student B | | | |
|  |  |  | Non-Academic | Teaching | Research | Total |
| Advisor's General Preference | Non-Academic | Count | **10** | 0 | 2 | 12 |
|  |  | % | **83.3%** | 0.0% | 16.7% | 100% |
|  | Teaching | Count | 1 | **1** | 2 | 4 |
|  |  | % | 25.0% | **25.0%** | 50.0% | 100% |
|  | Research | Count | 35 | 20 | **63** | 118 |
|  |  | % | 29.7% | 16.9% | **53.4%** | 100% |
|  | Total | Count | 46 | 21 | 67 | 134 |
| Advisor's Perceived Student B Preference | Non-Academic | Count | **45** | 2 | 9 | 56 |
|  |  | % | **80.4%** | 3.6% | 16.1% | 100% |
|  | Teaching | Count | 0 | **11** | 0 | 11 |
|  |  | % | 0.0% | **100.0%** | 0.0% | 100% |
|  | Research | Count | 4 | 5 | **54** | 63 |
|  |  | % | 6.3% | 7.9% | **85.7%** | 100% |
|  | Total | Count | 49 | 18 | 63 | 130 |

**Note.** % refers to percentage within general advisor preference (for top) and within advisor’s perceived Student B preference (for bottom). The top cross-tabulation indicates that there is a strong correspondence between the advisors’ general preferences and the advisors’ preferences for Student B, χ^2^ (4, *N* = 134) = 14.41, *p* =.006. Examining the diagonal (bolded) indicates that within the category of advisors’ general preferences, there is concordance for non-academic, and modest correspondence for teaching and research. The bottom cross-tabulation indicates strong concordance between the advisors’ perceived Student B preference and the advisors’ preference for Student B, χ^2^ (4, *N* = 130) = 147.93, *p* < .001. Examining the diagonal (bolded) indicates that within the category of Advisors’ Perceived Preference of Student A, there is very strong concordance.

| **Table 2** |  |  |  |  |  |  |
| --- | --- | --- | --- | --- | --- | --- |
| *Rank of Self and Perceived Student C (Third Most Senior PhD Student) Career Preference* | | | | | | |
|  |  |  | Advisor's Preference for Student C (Ranking) | | | |
|  |  |  | Non-Academic | Teaching | Research | Total |
| Advisor's General Preference (Ranking) | Non-Academic | Count | **4** | 2 | 2 | 8 |
|  |  | % | **50.0%** | 25.0% | 25.0% | 100% |
|  | Teaching | Count | 0 | **1** | 1 | 2 |
|  |  | % | 0.0% | **50.0%** | 50.0% | 100% |
|  | Research | Count | 28 | 14 | **32** | 74 |
|  |  | % | 37.8% | 18.9% | **43.2%** | 100% |
|  | Total | Count | 32 | 17 | 35 | 84 |
| Advisor's Perceived Student C Preference (Ranking) | Non-Academic | Count | **18** | 1 | 4 | 23 |
|  |  | % | **78.3%** | 4.3% | 17.4% | 100% |
|  | Teaching | Count | 1 | **9** | 1 | 11 |
|  |  | % | 9.1% | **81.8%** | 9.1% | 100% |
|  | Research | Count | 6 | 7 | **34** | 47 |
|  |  | % | 12.8% | 14.9% | **72.3%** | 100% |
|  | Total | Count | 25 | 17 | 39 | 81 |

**Note.** % refers to percentage within general advisor preference (for top) and within advisor’s perceived Student C preference (for bottom). The top cross-tabulation indicates that there is no significant correspondence between the advisors’ general preferences and the advisors’ preferences for Student C, χ^2^ (4, *N* = 84) = 2.70, *p* =.61. Examining the diagonal (bolded) indicates that within the category of advisors’ general preferences, there is modest correspondence. The bottom cross-tabulation indicates strong concordance between the advisors’ perceived Student C preference and the advisors’ preference for Student C, χ^2^ (4, *N* = 81) = 60.40, *p* < .001. Examining the diagonal (bolded) indicates that within the category of Advisors’ Perceived Preference of Student A, there is very strong concordance

| **Table 3** | |  |  |  |  |  |  | |
| --- | --- | --- | --- | --- | --- | --- | --- | --- |
| *Advisor's preferences for different career options for Students B and C as a function of their general preferences for those options and the perceived preference the advisor had for Student B and C* | | | | | | | |  |
|  | Career Option |  | *b* | Std. Error | 𝛽 | *t* | *p* | |
| Advisor’s Preference for Student B | Non-Academic | Constant | 1.32 | .38 |  | 3.44 | .001 | |
|  |  | General Rating | .27 | .05 | .24 | 4.90 | <.001 | |
|  |  | Perceived Student B Rating | .59 | .04 | .67 | 13.44 | <.001 | |
|  | Teaching | Constant | .57 | .39 |  | 1.48 | .14 | |
|  |  | General Rating | .23 | .06 | .19 | 3.99 | <.001 | |
|  |  | Perceived Student B Rating | .66 | .05 | .71 | 14.64 | <.001 | |
|  | Research | Constant | .88 | .94 |  | .93 | .35 | |
|  |  | General Rating | .12 | .10 | .06 | 1.18 | .24 | |
|  |  | Perceived Student B Rating | .72 | .06 | .68 | 12.36 | <.001 | |
| Advisor’s Preference for Student C | Non-Academic | Constant | 1.55 | .65 |  | 2.41 | .02 | |
|  |  | General Rating | .20 | .08 | .16 | 2.36 | .02 | |
|  |  | Perceived Student C Rating | .62 | .06 | .67 | 9.92 | <.001 | |
|  | Teaching | Constant | .80 | .45 |  | 1.80 | .08 | |
|  |  | General Rating | .23 | .07 | .18 | 3.43 | .001 | |
|  |  | Perceived Student C Rating | .69 | .05 | .77 | 14.69 | <.001 | |
|  | Research | Constant | -.67 | 1.43 |  | -.47 | .64 | |
|  |  | General Rating | .27 | .15 | .13 | 1.83 | .07 | |
|  |  | Perceived Student C Rating | .71 | .08 | .62 | 8.59 | <.001 | |

We conducted three regression analyses for Students B and C, where the outcome variable was advisor’s preferences for them to pursue each career option. The two predictors were advisors’ general preference and their perceived students’ preferences, all on the continuous scale. In each regression, the strength of the perceived student preference was much stronger (Student B: non-academic standardized *ß* = .67 vs. .24; teaching standardized *ß* = .66 vs. .23; research standardized *ß* = .72 vs. .12; Student C: non-academic standardized *ß* = .67 vs. .16; teaching standardized *ß* = .77 vs. 18; research standardized *ß* = .62 vs. .13).

| **Table 4** | | | | |
| --- | --- | --- | --- | --- |
| *Advisors’ Demographic Characteristics* | | |  |  |
| Characteristics | | |  | N(%) |
| Age M(SD) | | | 49.1(11.6) |  |
| Years in Professoriate | | | 16.2(12.1) |  |
| School / Field of Study | | |  |  |
|  | UC - Santa Barbara | |  | 177(64.6) |
|  | Engineering | Computer Science; Electrical and Computer Engineering; Technology Management Program | | 15(8.5) |
|  |  | Materials; Mechanical Engineering | | 13(7.3) |
|  | Life Sciences | Bio-Molecular Science and Engineering; Chemical Engineering | | 6(3.4) |
|  |  | Earth Science; Geography | | 17(9.6) |
|  |  | Ecology, Evolution and Marine Biology; Molecular, Cellular and Developmental Biology | | 15(8.5) |
|  |  | Environmental Studies; Environmental Science and Management | | 10(5.7) |
|  | Math | Mathematics; Statistics and Applied Probability | | 18(10.2) |
|  | Physical Sciences | Chemistry; Bio-Chemistry; Physics | | 17(9.6) |
|  | Social Sciences | Anthropology; Linguistics | | 12(6.8) |
|  |  | Dynamical Neuroscience; Psychological and Brain Sciences; Sociology | | 22(12.4) |
|  |  | Economics; Communication | | 18(10.2) |
|  | Other/ Unspecified |  |  | 14(7.9) |
|  | UC - Merced | |  | 97(35.4) |
|  | Social Sciences | Anthropology; Cognitive and Information Sciences; Psychology | | 20(20.6) |
|  |  | Political Science, Public Health, Sociology | | 13(13.4) |
|  | Engineering | Applied Mathematics; Electrical Engineering; Computer Science | | 11(11.3) |
|  |  | Materials and Biomaterials Sciences Engineering; Mechanical Engineering | | 10(10.3) |
|  | Life Sciences | Biological Engineering; Environmental Systems; Quantitative Systems Biology (QSB) | | 18(18.6) |
|  | Physical Sciences | Chemistry; Chemical Biology; Physics | | 17(17.5) |
|  | Other / Unspecified |  |  | 8(8.2) |
| Missing | |  |  | 27 |
| Gender | |  |  |  |
|  | Male | |  | 165(60.7) |
|  | Female | |  | 104(38.6) |
|  | Other | |  | 2(0.7) |
|  | Missing |  |  | 29 |
| Race | |  |  |  |
|  | Asian / Asian-American | |  | 31(11.7) |
|  | Black / African-American | |  | 3(1.1) |
|  | Hispanic / Latino-American | |  | 21(7.9) |
|  | Multi-Racial | |  | 10(3.2) |
|  | Native American | |  | 1(0.4) |
|  | Other | |  | 12(3.8) |
|  | White / Caucasian American | |  | 188(70.7) |
|  | Missing |  |  | 35 |
| Professor Status | | |  |  |
|  | Assistant Professor | |  | 75(27.4) |
|  | Associate Professor | |  | 51(18.6) |
|  | Full Professor | |  | 144(52.6) |
|  | Other | |  | 4(1.5) |
|  | Missing |  |  | 27 |
| US Born | | | | |
|  | U.S. Born | |  | 176(64.9) |
|  | Non-U.S. Born | |  | 95(35.1) |
|  | Missing |  |  | 30 |
| College Generation Status | | |  |  |
|  | First-Generation College Student | |  | 61(22.6) |
|  | Continuing-Generation College Student | | | 209(77.4) |
|  | Missing | | | 31 |

Note that % excludes missing cases
